# Evaluation and comparison of multi-omics data integration methods for cancer subtyping

**DOI:** 10.1371/journal.pcbi.1009224

**Published:** 2021-08-12

**Authors:** Ran Duan, Lin Gao, Yong Gao, Yuxuan Hu, Han Xu, Mingfeng Huang, Kuo Song, Hongda Wang, Yongqiang Dong, Chaoqun Jiang, Chenxing Zhang, Songwei Jia

**Affiliations:** 1 School of Computer Science and Technology, Xidian University, Xi’an, China; 2 Department of Computer Science, The University of British Columbia Okanagan, Kelowna, British Columbia, Canada; University of Calgary, CANADA

## Abstract

Computational integrative analysis has become a significant approach in the data-driven exploration of biological problems. Many integration methods for cancer subtyping have been proposed, but evaluating these methods has become a complicated problem due to the lack of gold standards. Moreover, questions of practical importance remain to be addressed regarding the impact of selecting appropriate data types and combinations on the performance of integrative studies. Here, we constructed three classes of benchmarking datasets of nine cancers in TCGA by considering all the eleven combinations of four multi-omics data types. Using these datasets, we conducted a comprehensive evaluation of ten representative integration methods for cancer subtyping in terms of accuracy measured by combining both clustering accuracy and clinical significance, robustness, and computational efficiency. We subsequently investigated the influence of different omics data on cancer subtyping and the effectiveness of their combinations. Refuting the widely held intuition that incorporating more types of omics data always produces better results, our analyses showed that there are situations where integrating more omics data negatively impacts the performance of integration methods. Our analyses also suggested several effective combinations for most cancers under our studies, which may be of particular interest to researchers in omics data analysis.

## Author summary

Cancer is one of the most heterogeneous diseases, characterized by diverse morphological, phenotypic, and genomic profiles between tumors and their subtypes. Identifying cancer subtypes can help patients receive precise treatments. With the development of high-throughput technologies, genomics, epigenomics, and transcriptomics data have been generated for large cancer patient cohorts. It is believed that the more omics data we use, the more accurate identification of cancer subtypes. To examine this assumption, we first constructed three classes of benchmarking datasets to conduct a comprehensive evaluation and comparison of ten representative multi-omics data integration methods for cancer subtyping by considering their accuracy, robustness, and computational efficiency. Then, we investigated the influence of different omics data and their various combinations on the effectiveness of cancer subtyping. Our analyses showed that there are situations where integrating more omics data negatively impacts the performance of integration methods. We hope that our work may help researchers choose a proper method and an effective data combination when identifying cancer subtypes using data integration methods.

## Introduction

Cancer is a complex, heterogeneous, and serious disease extremely harmful to human health. Research on cancer has been going on for decades. With the continuous development and the declining cost of high-throughput technology, international collaborative projects, such as TCGA, ICGC, and CCLE, have generated and collected a large number of different omics data of the same cohort of cancer patients at different levels [1], including genome, epigenome, transcriptome, metabolome, and proteome. It has been accepted that different levels of biological data collectively affect and regulate multiple biological processes, and provide more reliable information for the formation and promotion of complex diseases [2]. As a powerful and valuable approach to utilizing different types of genomic data, multi-omics data integration has attracted much recent interest in the field of bioinformatics.

Applying data integration methods to cancer analysis has three main goals: understanding the molecular mechanism of cancer, clustering disease samples, and predicting an outcome, such as survival or therapy efficacy [3]. One of the most important tasks is the identification of molecular cancer subtypes, i.e. groups of patients with common biological characteristics or clinical phenotypes such as survival time and drug response. The treatments of different cancer patients are highly dependent on their specific subtypes [4]. By using data integration strategies and considering different levels of information, cancer subtypes can be identified from macro perspectives so that patients can get more accurate diagnoses and treatments [5].

**Fig 1:**
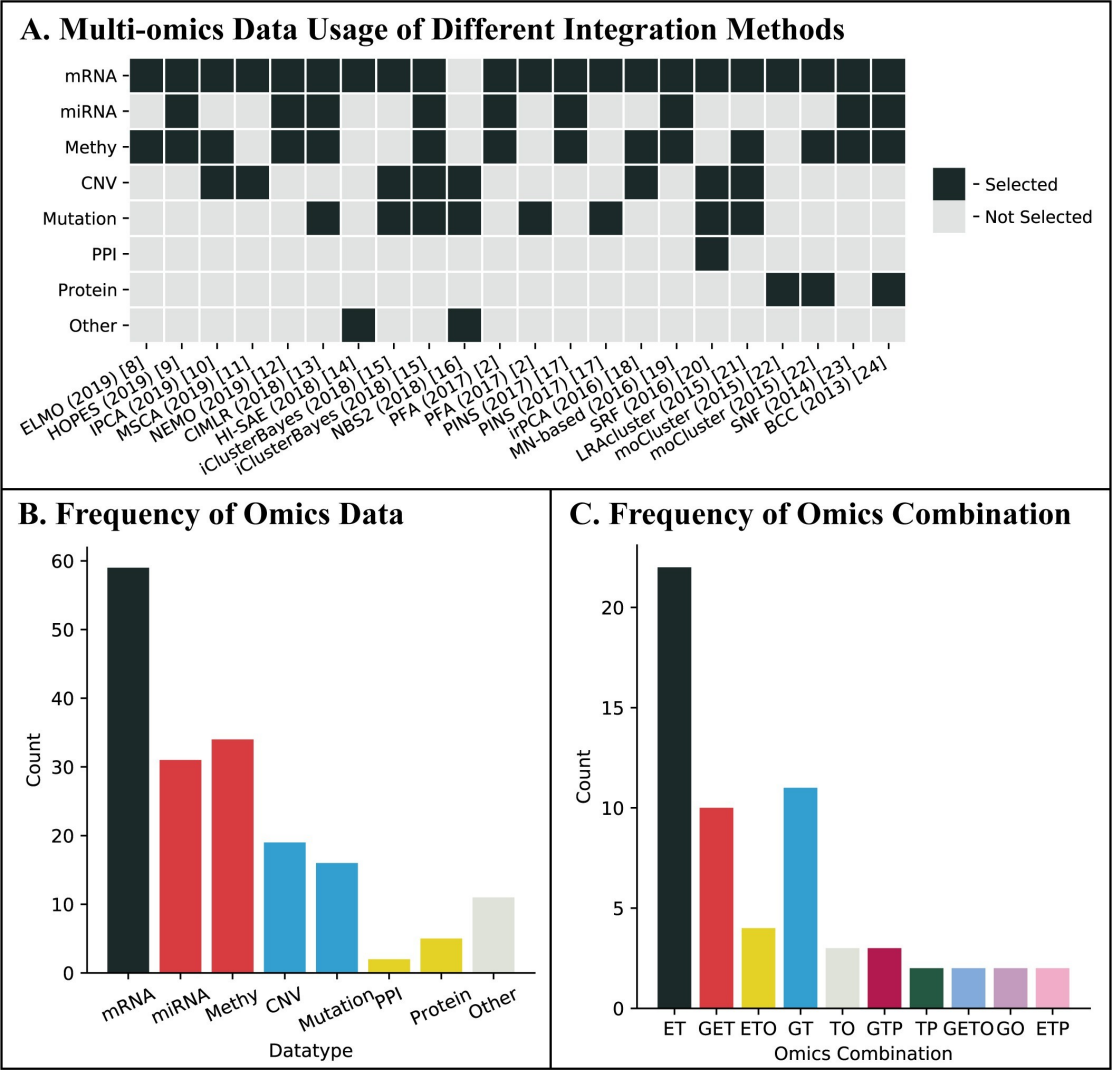
Summary of the data type selection of existing integration methods. (A) Multi-omics data usage of different integration methods. (B) The usage frequency of each omic data type. We use “Methy”, “mRNA”, “miRNA” and “protein” to represent DNA methylation, mRNA expression, miRNA expression, and protein expression, respectively. (C) The usage frequency of different omics combinations. We use “G”, “E”, “T”, “P” and “O” to represent genomics, epigenomics, transcriptomics, proteomics, and others, respectively.

In recent years, many computational integration methods for cancer subtyping have been proposed. To choose among these methods, practitioners usually face two inevitable problems. The first problem is about how to compare the performance among these methods and the second problem is related to the selection of available data types to integrate in order to achieve the best possible results. The first problem is due to the lack of gold standards and consistent performic criteria [6], and the fact that different datasets and evaluation metrics were used when different methods were proposed. To understand and demonstrate the crucial need for addressing the second problem of data type selection, we surveyed 58 integration methods for cancer subtyping proposed from 2009 to 2019, and the result is summarized in Fig 1 where gene expression is treated as the same as mRNA expression and miRNA expression is placed into the group of epigenome based on observations from [7]. We summarized part of these 58 integration methods with the omics data they used in Fig 1A, and we can see, the data combinations used in these methods [2,8–24] are significantly inconsistent. For example, Fig 1B shows that while the mRNA expression data were used by 56 of the 58 methods, each of the other data types was only used by at most nearly half of these methods. Regarding the combinations of data types used in these methods, we also observed a significant variation among these methods (Fig 1C). We shall note that in most of the papers proposing these methods, there was not much effort to discuss why a particular set of data types were selected from either computational or biological perspective.

In some recent studies on multi-omics integration methods, efforts have been given to understand the above two problems and most of the studies focused on the first problem on performance comparison and benchmarked the methods under only one performance criterion. Treating these subtyping methods as methods for unsupervised clustering, Chauvel et al. [25] evaluated iCluster [26], moCluster [22], iNMF [27], JIVE [28], MDI [29], and BCC [24] by using simulated datasets to study whether the method could recover the clusters they constructed and the consistency between simulated and estimated clusters using Adjusted Rand Index (ARI). On BRCA real datasets with true labels and subtypes markers, similar, but less sufficient, comparisons were conducted. Rappoport et al. [30] benchmarked LRAcluster [21], PINS [17,31], SNF [23], rMKL-LPP [4], MCCA [32], MultiNMF [33], and iClusterBayes [15] using 10 real cancer datasets by survival analysis and clinical parameters enrichment. The experiments were well designed and the analysis was interesting. However, the evaluation was still limited as it focused on a single clinical-based accuracy criterion.

To the best of our knowledge, only two studies [34,35] considered the second problem on the significance of the selection and combination of the data types. As both studies used different data types for different diseases (such as BXD, Platelet, and BRCA), it is not clear if the insights obtained are useful for cancer subtyping. This is particularly true for the work by Pierre-Jean [35] where different data type combinations were not considered at all on real disease datasets. The evaluation in these two studies is also limited in that the datasets generated or collected all had true labels and a clustering-based performance metric was used.

In this study, we constructed three classes of benchmarking datasets by integrating all the possible combinations of four different types of multi-omics data of nine different cancers. Using these datasets, we evaluated and compared the performance of ten representative integration methods for cancer subtyping by taking into consideration their clustering accuracy as well as their clinical significance. Based on the experiment results, we further studied the influence of different omics data and their various combinations on the effectiveness of data integration methods for cancer subtyping.

## Materials and methods

In this section, we briefly review existing integration methods and discuss in detail every process of our experiments, including data pre-processing, dataset construction, integration and subtyping, and performance evaluation.

### Selection of integration methods

Researchers have been committed to developing computational multi-omics data integration methods for cancer subtyping using different strategies or techniques. Previous studies have classified the current integration methods into several categories based on different criteria, including clustering of clusters [24,29], transformation-based approaches [4,36], and deep learning-based approaches [14,37–41], which we summarize in Table 1. It is worth noting that in these categorizations, a particular method may belong to multiple categories.

**Table 1:** Summary of previous studies.

Study	Categories		Methods
Wang et al. (2016) [42]	direct integrative clustering		[21,28,43–46]
	clustering of clusters		[23,24,29,47]
	regulatory integrative clustering		[48]
Tini et al. (2017) [34]	multivariate		[32,49–57]
	concatenation-based		[21,22,26,28,58–61]
	transformation-based		[4,23,36,62–64]
Rappoport et al. (2018) [30]	early integration		[21,26,65]
	late integration		[17,47,66]
	intermediate integration	similarity-based	[4,23,67–73]
		dimension reduction	[21,28,32,33,45,50,53,58,74–80]
		statistical modeling	[15,24,26,29,43,48,81–87]
		deep learning-based	[86,87]
Chauvel et al. (2019) [25]	conceptual integration		[88]
	consensus clustering		[89]
	concatenation-based integration		[90,91]
	searching for common variations		-
	multi-omic pathway enrichment		[92,93]
Subramanian et al. (2020) [1]	multivariate		[4,22,27,28,58,59,94]
	similarity		[12,17,23]
	network		[48]
	fusion		[2,23,81]
	Bayesian		[21,24,26,29,43,48,81]

Aiming at a systematic evaluation of integration methods and data selection issues, in this work, we adopted the simple methodology-based division proposed by Bersanelli [3], where integration methods are grouped into two categories: network-based methods and statistics-based methods. We selected five representative network-based methods, Similarity Network Fusion (SNF) [23], Neighborhood based Multi-Omics clustering (NEMO) [12], Cancer Integration via Multikernel Learning (CIMLR) [13], Multi-View NMF (MultiNMF) [33], and Pattern Fusion Analysis (PFA) [2], and three widely used statistics-based integration methods, Low-rank Approximation Based Multi-omics Data Clustering (LRAcluster) [21], moCluster [22], and iClusterBayes [15]. We also selected an integrative framework Perturbation Clustering for Data Integration and Disease Subtyping (PINS) [17,31] in our study which cannot be placed in either of the two categories. As deep learning techniques have been widely used in the field of bioinformatics and achieved many successes, we added a deep learning-based method, Subtype-GAN [95], into our experiment. We notice that LRAcluster, PFA, and MultiNMF are not originally designed for cancer subtyping problems but these methods represent general frameworks for integrating multi-omics data, which can be used to conduct different downstream analysis including cancer subtyping and have good performances [1,11,12,30]. For evaluation and comparison of more integration methods, we included these three methods in this study. We briefly discuss the selected ten integration methods as follows.

1. **SNF** constructs sample-sample similarity networks, one for each type of omics data, where the nodes representing the samples and weighted edges between nodes representing the similarity between the samples. A message-passing process is then used to update the weights of similarity among multiple similarity networks on the same set of samples iteratively to make these networks more and more similar. The spectral clustering [96] method is used on the converged similarity network to identify cancer subtypes.
2. **NEMO** builds a similarity network between samples for each omics data and then modifies the similarity to relative similarity (RS) which is more comparable between omics. When integrating different omics data, NEMO simply averages RS in the different similarity networks for each pair of samples. Cancer subtypes are identified on the averaged similarity network using spectral clustering. NEMO can handle partial multi-omics datasets in the situation that each pair of samples has measurements in at least one common omics in which they are both measured.
3. **CIMLR** uses different Gaussian kernels to measure patient-to-patient distance and then learns weights for these multiple kernels in each data type. After estimating the number of clusters using the gap statistic, it combines the multiple kernels into a resulting similarity matrix with a block structure. K-means clustering is used on the resulting similarity matrix to identify cancer subtypes.
4. **MultiNMF** is a multi-view clustering algorithm based on nonnegative matrix factorization. It obtains a shared coefficient matrix as the common consensus data matrix by formulating a joint matrix factorization process with the constraint. The consensus data matrix is considered as a latent representation of the original data points reflecting the latent clustering structure shared by different views. K-means is used directly on this consensus matrix to obtain the cancer subtyping results.
5. **PFA** exacts local information of each biological dataset using a dimension reduction strategy. Based on an adaptive optimization method, it aligns local sample patterns into a global sample-spectrum. The clustering method K-means [97] is used on the sample-spectrum matrix to identify cancer subtypes.
6. **LRAcluster** uses a low-rank approximation based integrative probabilistic model to integrate different types of multi-omics data. The real-type, binary, and count-based data matrices are modeled as different distributions determined by a latent representation of the original data matrix. The objective function of LRAcluster is designed to be convex so that the global optimal can be found by the simple gradient-ascent algorithm. To clustering the samples, the K-means method is used on the latent representation matrix.
7. **moCluster** is based on a multitable multivariate analysis. It finds latent variables using sparse consensus PCA and determines the number of latent variables using permutation and elbow test. Cancer subtypes are identified using conventional clustering methods such as hierarchical clustering and K-means clustering method.
8. **iClusterBayes** uses different models for continuous, binary, and count data. It integrates different types of omics data by projecting them into a common low-dimensional integrated space, using the Bayesian latent variable regression model. K-means clustering is performed on the projected latent representation of the samples.
9. **PINS** uses its own perturbation clustering method to cluster each omics datasets and constructs connectivity matrices which will be merged into a combined similarity matrix subsequently. It uses similarity-based clustering methods such as uses hierarchical clustering [98], PAM [99], or Dynamic Tree Cut [100] to identify subtypes from the integrated similarity matrix.
10. **Subtype-GAN** is a deep learning-based multi-omics data integration approach for discovering cancer subtypes. It extracts features from each omics data by relatively independent layers and integrates different omics by feeding the extracted information to the same shared layer simultaneously. Consensus GMM clustering is used to predict cancer subtyping results.

### Omics data pre-processing

**Fig 2:**
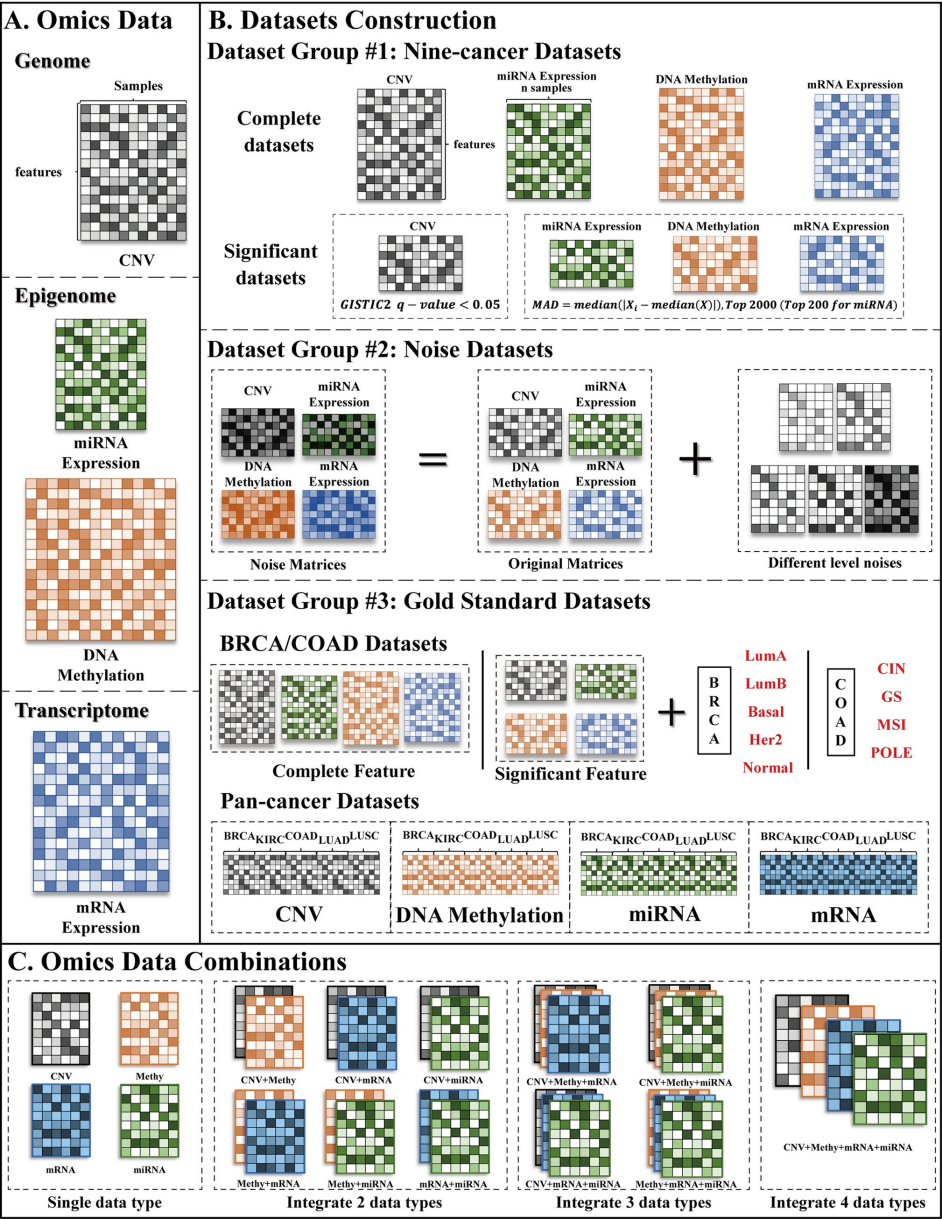
Data usage. (A) Omics data types we used in this study. The main color of each heatmap (matrix) represents one type of omics data, i.e. black represents CNV, green represents miRNA expression, orange represents DNA methylation, and blue represents mRNA expression. The shade of color in each heatmap is proportional to the different values. (B) Datasets construction. As TCGA group assigned BRCA and COAD patients into five (i.e. “LumA”, “LumB”, “Basal”, “Her2”, and “normal”) and four (i.e. “CIN”, “GS”, “MSI”, and “POLE”) subtypes, respectively, in BRCA and COAD gold standards datasets of Dataset group #3, we use these assignments as gold standards of BRCA and COAD patients. (C) Omics data combinations. We use “Methy”, “mRNA”, and “miRNA” to represent DNA methylation, mRNA expression, and miRNA expression, respectively.

The Cancer Genome Atlas Program (TCGA) has collected a large number of different types of omics data from more than 30 types of cancers. In this study, four types of omics data were chosen, including copy number variation in genome level, DNA methylation and miRNA expression in epigenome level, and mRNA expression in transcriptome level (Fig 2A). To decide the cancer types to be used in our studies, we focused on those cancers which had a sufficient number of samples of the four types of omics data in the TCGA collection and had been used in previous studies on cancer subtyping. As a result, nine common cancers were chosen, including Adrenocortical Carcinoma (ACC), Breast Invasive Carcinoma (BRCA), Colon Adenocarcinoma (COAD), Kidney Renal Papillary Cell Carcinoma (KIRP), Kidney Renal Clear Cell Carcinoma (KIRC), Liver Hepatocellular Carcinoma (LIHC), Lung Adenocarcinoma (LUAD), Lung Squamous Cell Carcinoma (LUSC), and Thymoma (THYM).

For the mRNA and the miRNA expression data, we downloaded the level-three data of HTSeq-FPKM data and BCGSC miRNA Profiling miRNA-Seq data from TCGA. For the DNA methylation data, we chose Illumina Human Methylation 450 level-three data from TCGA. We selected the promoter-associated probes and mapped them to each gene as LRAcluster did. The promoter regions were based on Yang’s previous study [101,102]. When evaluating the methylation level of each gene, we observed that multiple probes might map to the same gene. Therefore, we used the median value of these probes to represent the methylation level of that gene. For CNV data, we also mapped CNV regions to each gene and found that a gene might span multiple CNV regions. In this case, we took the same strategy to evaluate the copy number level of each gene using the median value of its corresponding CNV regions.

For each type of omic data, we first filtered the features that had more than 20% missing values across all patients and filtered the samples that had more than 20% missing values across all features as SNF did. Then, we selected the samples that belonged to the same patient cohort for four omics datasets. We used the K-nearest neighbor method to impute the missing values [103]. Since batch effects had been reported among most high-throughput technologies [104] which might disturb the experiment, we removed the batch effects using the ComBat function [105] to avoid the disturbance, and the essential batch numbers were extracted from the clinical information downloaded from TCGA. Finally, we normalized each dataset by calculating z-scores to eliminate the differences due to the use of different scales in these datasets.

### Benchmarking dataset construction

To make a comprehensive evaluation and comparison, we constructed three different classes of benchmarking datasets using four types of omics data of nine cancers. Fig 2B shows the details of dataset construction.

1. **Dataset group #1: Nine-cancer datasets.** Dataset group #1 considers all four types of omics data for each of nine cancers. There are also two versions for each cancer dataset. The first version of each dataset contains samples with all features after the data processing step, which is denoted as the complete dataset. The second version of each dataset contains samples that only have significant features, which is called the significant dataset. We explain in the following how significant features are determined. For the mRNA expression and the DNA methylation data, we adopted the median absolute deviation (MAD) to select the top 2,000 most-variable features as iClusterPlus [43] did. For the miRNA expression data, we calculated MAD for each miRNA and selected the top 200 miRNAs because the number of miRNAs was too small. As GISTIC2 [106] identified genomic regions that were significantly gained or lost across a set of tumors, therefore, for the CNV data, we selected the genes in significantly gained or lost regions (q-value < 0.05) reported in Broad Institute for each cancer. We notice that the scales of the significant datasets are much smaller than those of the complete datasets since only approximately 10% of features of each omic data are reserved.
2. **Dataset group #2: Noise datasets.** We used the significant datasets of BRCA and COAD in Dataset group #1 as the original datasets. Noise datasets were constructed by adding Gaussian noises with five different levels to each of the original datasets. The mean of the Gaussian noise is the same as the original dataset, and the variance is 0.5, 1, 2, 3, and 4 times of the original one, respectively.
3. **Dataset group #3: Gold standards datasets.** There are two parts of Dataset group #3. The first part includes the BRCA and COAD datasets with true labels. Although there is no gold standard for cancer subtyping, subtypes of BRCA and COAD patients have been analyzed by the TCGA group in a previous study [107], and we considered these subtypes as the true labels. For each of these two cancers, we used the samples studied in [107] that are in both of the complete and significant datasets in Dataset group #1 to construct the first part of the gold standard datasets. The second part consists of Pan-cancer datasets. Using the complete datasets of BRCA, COAD, KIRC, LUAD, and LUSC in Dataset group #1, we identified the common features of all five cancers for each omic data and combined the omic datasets of the five cancers together with those common features to construct Pan-cancer datasets. The cancer labels were considered as true labels.

Previous studies on integrative cancer analysis have used different combinations of multi-omics data, among which mRNA expression + miRNA expression and mRNA expression + miRNA expression + DNA methylation are the two most used combinations. Because of the inconsistency of the data combinations used in previous studies and the lack of a common guideline, we enumerated all the possible combinations of the four types of omics data. As Fig 2C shows, there are six two-omic combinations, four three-omic combinations, and one four-omic combination, giving a total of 11 possible combinations. We used each of these combinations to construct the three classes of benchmarking datasets illustrated above and these datasets were all used in our experiments.

### Omics data integration and subtyping

We used the packages or codes provided by the authors of these ten representative integration methods to obtain the subtyping results on each data combination in the whole benchmarking datasets. Table 2 lists the details of these packages. We also did some improvements to these packages, including the parallelization of LRAcluster, running iClusterBayes in parallel without operating system limitation, and removing PFA’s limitation on the number of data types it could handle. Details of these technical improvements can be found in our recent paper [6] (https://github.com/GaoLabXDU/CEPICS). Since the LRAcluster, PFA, and MultiNMF package only output an integrated sample-feature matrix rather than clustering (subtyping) results, we used the K-means clustering with 300 iterations on the sample-feature matrices to obtain stable subtyping results.

**Table 2:** Details of packages or codes used in this work.

Method	Package name/Source code	Version	Language	Platform	Resource Download Link
SNF	SNFTool	2.3.0	R/MATLAB	CRAN/-	http://compbio.cs.toronto.edu/SNF/
NEMO	Source code	-	R	-	https://github.com/Shamir-Lab/NEMO
CIMLR	CIMLR	1.0.0	R/MATLAB	-/-	https://github.com/danro9685/CIMLR
MultiNMF	Source code	-	MATLAB	-	http://jialu.info/code/Code_multiNMF.zip
PFA	Source code	-	MATLAB	-	http://sysbio.sibcb.ac.cn/sysbio/cb/chenlab/software.htm
LRAcluster	LRAcluster	1.0	R	-	http://lifeome.net/software/lracluster/
moCluster	mogsa	1.22.1	R	Bioconductor	
iClusterBayes	iClusterPlus	1.20.0	R	Bioconductor	
PINS	PINSPlus	2.0.0	R	CRAN	
Subtype-GAN	Subtype-GAN	-	Python	-	https://github.com/haiyang1986/Subtype-GAN

### Performance evaluation

Using the subtyping results we obtained, we evaluated the performance of the ten integration methods. To make a comprehensive and reliable comparison, three performance criteria were used: the accuracy, the robustness, and the computational efficiency.

1. **Accuracy**. The accuracy is the most important factor for users to choose a method. Accurate results may help researchers find new cancer subtypes and make precise treatments for different cohort patients. However, evaluating the accuracy of integration methods for cancer subtyping is challenging because of the lack of gold standards and ground truth. We believe that in addition to the use of clustering-based metrics, subtyping results should also be evaluated using clinically-relevant metrics to make sure that good subtyping results capture significant differences in clinical features. Therefore, we evaluated and compared the accuracy of the ten methods by taking into consideration both of the clustering accuracy and the clinical significance. For unlabelled datasets, we used the log-rank test p-value and silhouette coefficient to evaluate the subtyping results. The p-value of the log-rank test is the most wildly used metric that represents the extent of the significance of different cohort patients on survival time from the clinical aspect [108–111]. The silhouette coefficient [112] measures the similarity between a sample and its classified subtype in comparison to the samples in the other subtypes to determine how appropriately samples in a dataset have been clustered. For labelled datasets, we used precision, normalized mutual information (NMI), adjusted rand index (ARI), and F-measure to evaluate the degree of agreement between the subtyping results obtained by these methods and the true labels. Precision is the percentage of samples that are classified into the correct subtype. When calculating the precision of each subtyping result, it was necessary to label each cluster with its corresponding subtype. We adopted a heuristic strategy that labelled a cluster as the subtype with the largest number of matches.
2. **Robustness**. Due to the limitations of sequencing techniques and experiment conditions, different levels of noise exist in the omics data. When we integrate multi-omics data, the effects of noise may get worse and cause inaccurate subtyping results. It is, therefore, necessary and important to take into consideration the robustness of integration methods. We evaluated the robustness of each method by calculating NMI and ARI between the results of original datasets and the corresponding noise datasets disturbed by different levels of noise.
3. **Computational Efficiency**. Typical biological datasets are known as small-sample-high-dimension datasets in that the number of samples might not be very huge, but the number of features associated with a sample is normally large. In most omics datasets, there are normally a few hundred samples, while each sample typically has thousands of features. For example, the original COAD methylation dataset has 291 samples, but a sample has over 450 thousand features. Datasets with such characteristics pose a great challenge for integration methods since the running time of these integrated methods may become too high.

Using the experiment results on the accuracy of the integration methods we used on all possible combinations of the omics data types of the cancers, we further analyzed the influence of different omics data on the effectiveness of data integration in cancer subtyping. Moreover, to understand the inconsistency of the results reported in different previous studies of data integration methods for cancer subtyping, we studied the impact of different data combinations on the performance of the integration methods for eight of nine cancers and identified some data combinations that are effective for most of the eight cancers. We hope that our analysis and observations serve as useful guidance for the practice of using an integrated approach to cancer subtyping.

## Results

We evaluated each method in terms of its accuracy, robustness, and computational efficiency on the constructed benchmarking datasets. A comprehensive comparison of the performance of the ten integration methods was conducted, taking into consideration both their clustering accuracy and their clinical significance. We further analyzed the influence of different types of omics data and their combinations on cancer subtyping.

### Accuracy

As cancer subtyping results are generally obtained by unsupervised clustering, the determination of the number of cancer subtypes/clusters (denoted as *k*) is difficult and still an open question. We notice that, in these ten selected methods, iClusterBayes, SNF, PINS, NEMO, moCluster, CIMLR, and Subtype-GAN have their own criteria to estimate the best *k* from the user-specified range. For SNF, NEMO, and CIMLR, they estimate the best *k* automatically according to eigen-gaps or rotation cost. For PINS, it automatically evaluates the instability of different connectivity matrices to determine the best *k*. For iClusterBayes and moCluster, they calculate different metrics (deviance ratios and Bayesian information criterion values for iClusterBayes, and gap-statistic for moCluster) to generate a plot that is used to choose the best *k* by users manually. For Subtype-GAN, it uses Consensus GMM clustering to choose the best *k*. Therefore, we evaluate and compare the performance of all methods suggested and all possible number of clusters to make our experiments comprehensive. All ten integration methods were applied to each dataset in Dataset group #1 and #3, using each of the 11 possible combinations of the data types, which were summarized in Table 3. The calculations of Dataset group #1 and #3 were reported in S2 and S3 Files, respectively.

**Table 3:** Testing strategies and the datasets of the accuracy tests.

Dataset		Cancer	*k*	Evaluation Metrics
Dataset group #1: Nine-cancer datasets	Complete	BRCA; COAD; KIRC; LUAD; LUSC; ACC; KIRP; LIHC; THYM	2–8	Silhouette; Log-rank test
	Significant			
Dataset group #3: Gold standards datasets	Complete	BRCA; COAD	BRCA: 5 COAD: 4	Precision; F-measure; NMI; ARI
	Significant			
	Pan-cancer	-	5	

For Dataset group #1, as the true number of subtypes (i.e. *k*) was unknown, we first evaluated and compared the performance based on the number of clusters suggested by each method. As these methods require users to specify a maximum number of clusters (i.e. *k*-max), we set *k*-max to 8, which was the maximum number of subtypes commonly identified in cancer subtyping studies [15,17,23]. All the method-suggested *k* were listed in S1 Table in S1 File. Moreover, we also experimented with all possible values between 2 and 8.

**Fig 3:**
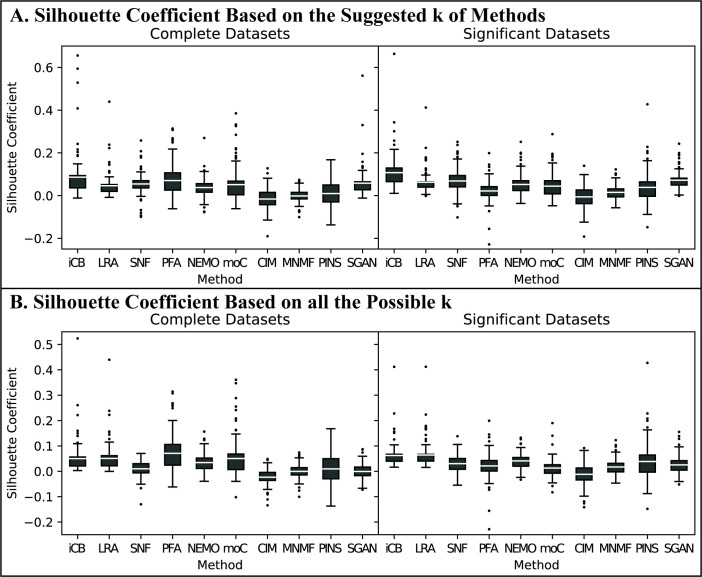
Clustering-based performance of Dataset group #1 Nine-cancer Datasets. We use “iCB”, “LRA”, “moC”, “CIM”, “MNMF”, and “SGAN” to represent iClusterBayes, LRAcluster, moCluster, CIMLR, MultiNMF, and Subtype-GAN respectively. (A) Silhouette coefficient based on the suggested *k* of methods. We set *k*-max as 8 and let each method suggest the best *k*. Each of the 11 data points in a box represents a silhouette coefficient of the subtyping results based on the method suggested *k* obtained by the corresponding method using one of the 11 possible combinations of data types. (B) Silhouette coefficient based on all the possible *k*. Each of the 11 data points in a box represents the average silhouette coefficient of the subtyping results from *k* = 2 to 8 obtained by the corresponding method using one of the 11 possible combinations of data types.

The clustering accuracy of the integration methods was measured by the silhouette coefficient. Note that silhouette coefficient can be calculated in the original space or in the integrated space. In this study, we calculated silhouettes using the concatenated input data matrices in the original space as the authors of PINS did. For the results using the method-suggested *k*, we found that iClusterBayes had the highest silhouette coefficient in most cancer datasets followed by Subtype-GAN and SNF (Fig 3A). For the results using all possible *k* (i.e., *k* = 2 to 8), iClusterBayes and LRAcluster performed the best (Fig 3B) followed by NEMO and PFA. SNF and Subtype-GAN performed worse compared to its performance of method-suggested *k*. We also calculated the silhouettes in the integrated space. The results were shown in S1 Fig in S1 File and the calculations were reported in S2 File.

**Fig 4:**
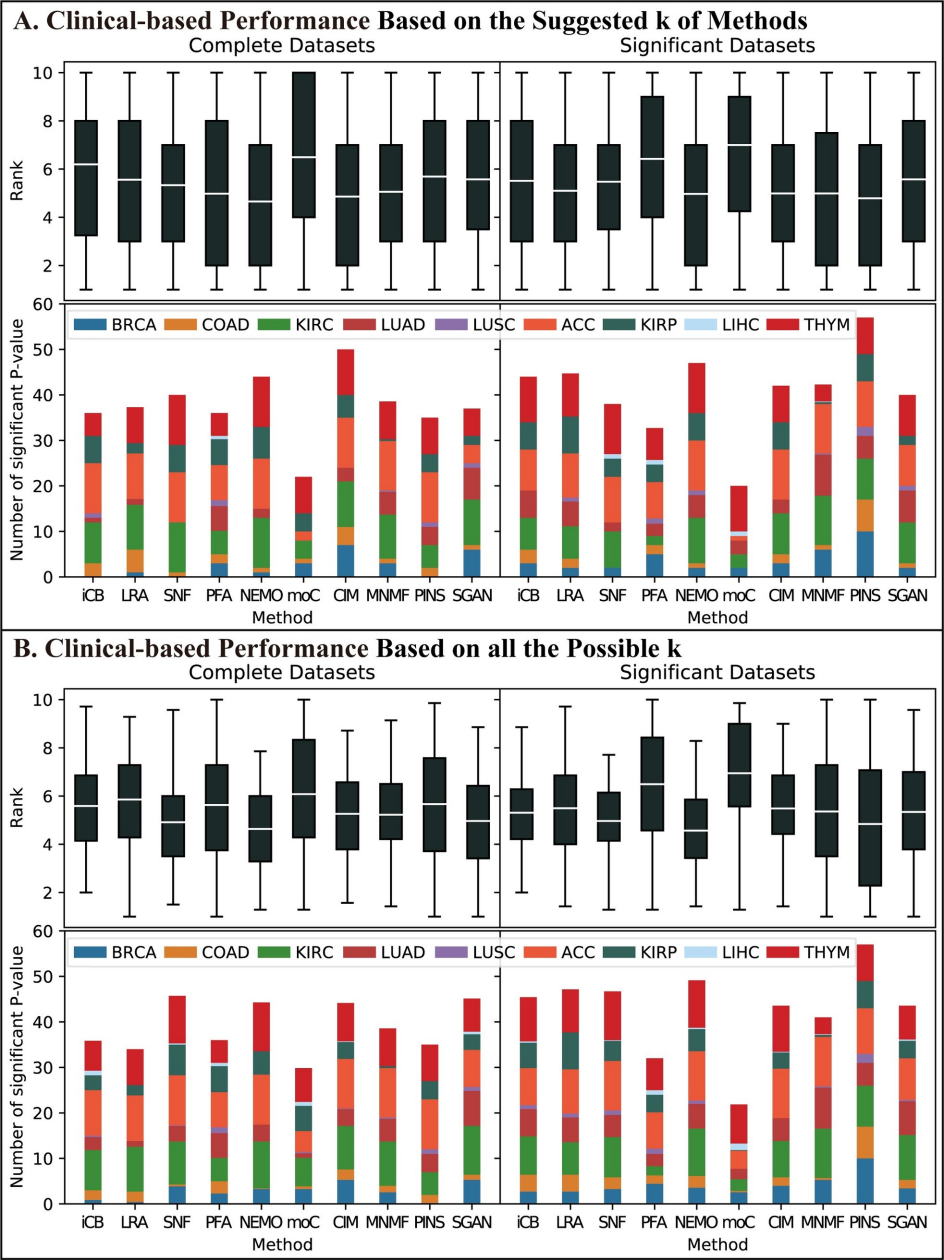
Clinical-based performance of Dataset group #1 Nine-cancer Datasets. The representations of the abbreviations are the same as those in Fig 3. We calculated the -log10(log-rank test p-value) of the subtyping results based on every possible *k*, combination, and cancer of each method. (A) Clinical-based performance based on the suggested *k* of methods. The upper plot shows the average ranking of the ability to cluster patients into clinically-significant subtypes of each method. Each data point in the box was calculated as follows. We fixed cancer and combination to rank the -log10(p-value) among all methods, which represented the ability of clustering patients into clinically-significant subtypes of each method using the current combination. Then each method had 11 (combinations) * 9(cancers) rankings which we used to compare these methods. The lower plot shows the cumulative number of significant p-values. We set 1.301 as the threshold which corresponded to 0.05 before the transformation to evaluate whether the current subtyping result had clinical significance and we counted the significant ones. (B) Clinical-based performance based on all the possible *k*. Two plots had the same meaning as (A) but the ways of calculation were a little different. Each data point in the box of the upper plot was calculated as follows. We fixed cancer, combination, and *k* to rank the -log10(p-value) among all methods. Therefore, each combination had 7 rankings corresponding to each possible *k*, and we then calculated the average of these 7 rankings to represent the ability of using the current combination. For the lower plot, we counted the number of significant p-values for each combination among all possible *k* and cumulated the average of each combination to draw the plot.

To evaluate the clinical significance of the integration methods, we downloaded the clinical information of these nine cancers from TCGA. Based on subtyping results, we calculated the log-rank test p-values and their rankings across methods as the performance metrics to evaluate the clinical significance. Fig 4A summarizes the clinical-based performance of the integration methods on the nine-cancer datasets in Dataset group #1 based on method-suggested *k*. We observed that NEMO and PINS had the best average ranking and identified more significant cancer subtypes than other methods. Fig 4B summarizes the performance based on all the possible *k*. NEMO and SNF performed the best on both two metrics. CIMLR, PINS, and MultiNMF performed well and similarly.

**Fig 5:**
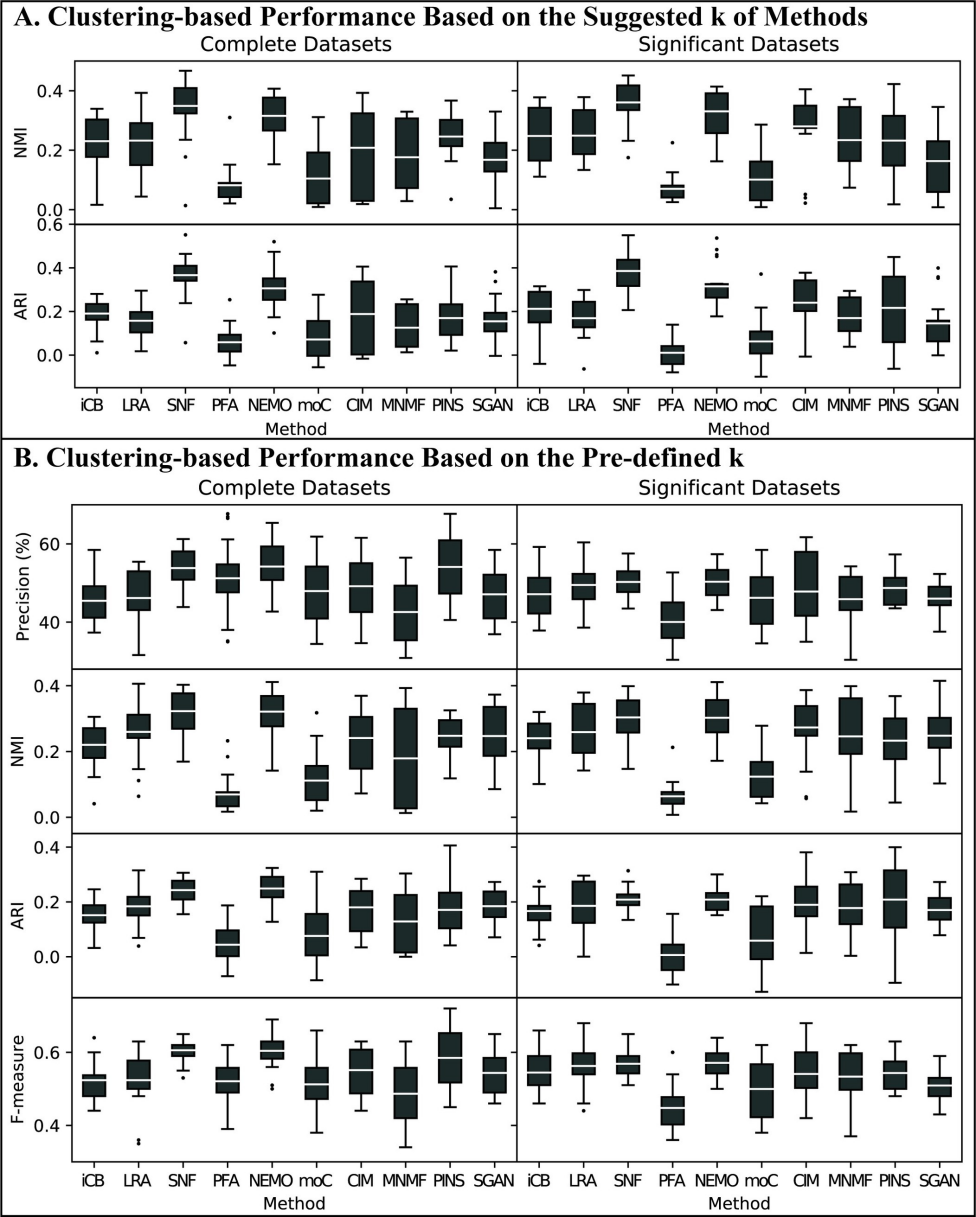
Clustering-based performance of Dataset group #3 Gold Standard Datasets. For each metric (i.e. precision, NMI, ARI, and F-measure) and each integration method, each data point in a box is a measurement of using one of the 11 data type combinations for both BRCA and COAD datasets, and the white line within the box indicates the mean value of the results. (A) Clustering-based performance of gold standard datasets based on the suggested *k* of methods. We set *k*-max as 8 and let each method suggest the best *k*. The performance of the method suggested *k* was used to evaluate and compared. To the three methods that cannot suggest best *k*, we clustered BRCA and COAD samples into 5 and 4 clusters, respectively. (B) Clustering-based performance of gold standard datasets based on the pre-defined *k*. As the true labels of samples and the number of clusters are known in Dataset group #3, we clustered BRCA and COAD samples into 5 and 4 clusters, respectively, and calculated the clustering-based metrics to evaluate and compare the performance of the integration methods.

For Dataset group #3, we also compared the performance based on method-suggest *k* first. We listed the suggested *k* of each method in S2 Table in S1 File. Since LRAcluster, PFA and MultiNMF cannot suggest *k*, we clustered BRCA and COAD samples into 5 and 4 clusters for these methods, respectively. NMI and ARI were used to evaluate clustering-based accuracy. We found that SNF and NEMO had the best average performance for both metrics and for both complete and significant datasets. CIMLR, PINS, and LRAcluster had comparable performance on NMI while iClusterBayes, CIMLR and Subtype-GAN had comparable performance on ARI (Fig 5A).

As the true labels of samples and the number of clusters were known in Dataset group #3, we next clustered BRCA, COAD, and Pan-cancer samples into 5, 4, and 5 clusters, respectively. Precision, F-measure, NMI, and ARI were used to evaluate the clustering accuracy. We obtained similar observations that SNF and NEMO had the best average performance followed by CIMLR and LRAcluster (Fig 5B).

**Fig 6:**
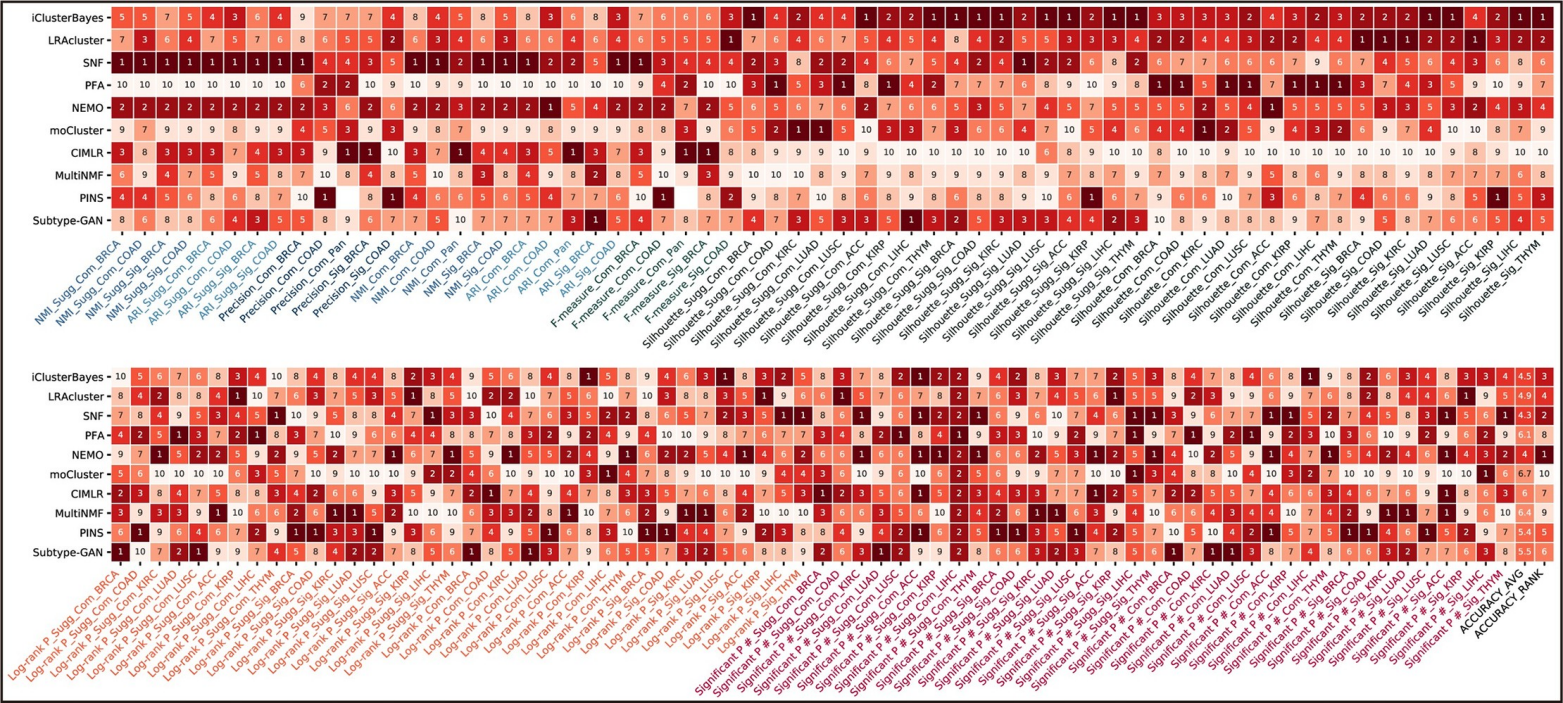
Accuracy Rank Table. The rank items listed under the table include the information of metrics, datasets, cancer, whether based on the method suggested *k*, which are connected by underlines. “Sugg” represents the current test is based on the method suggested *k*. “Com” and “Sig” represent the complete and significant datasets, respectively.

To get an overall ranking of these integration methods, we aggregated the performance measurements of them over all datasets and all performance metrics using the following formula:

$$Avg\_Rank=\frac{\sum R_{NMI\_Suggest}+\sum R_{ARI\_Suggest}+\sum R_{Precision}+\sum R_{NMI}+\sum R_{ARI}+\sum R_{F-measure}+\sum R_{Silhouette\_Suggest}+\sum R_{Silhouette}}{2(Item_{NMI\_Suggest}+Item_{ARI\_Suggest}+Item_{Precision}+Item_{NMI}+Item_{ARI}+Item_{F-measure}+Item_{Silhouette\_Suggest}+Item_{Silhouette})}+\frac{\sum Rank_{Log-rank\_Suggest}+\sum Rank_{Significant\#\_Suggest}+\sum Rank_{Log-rank}+\sum Rank_{Significant\#}}{2(Item_{Log-rank\_Suggest}+Item_{Significant\#\_Suggest}+Item_{Log-rank}+Item_{Significant\#})}$$

where *R*_(∙)_ is the ranking of a method as evaluated with a specific metric and a specific dataset, while *Item*_(∙)_ is the number of the datasets used for a specific metric. The rationale behind the above formula is that a good method should be accurate under both clustering-based metrics and clinical-based metrics. As shown in Fig 6, NEMO has the best overall performance followed by SNF, iClusterBayes, and LRAcluster. The aggregated rankings of the other six methods are comparable.

### Robustness

We believe that robust methods are those that have a reasonable degree of resilience to noise in the data. Under this assumption, we used Dataset group #2 to study how quickly the subtyping results of these methods change as higher and higher levels of noise are introduced into the data. In this set of experiments, for each method *i*, we obtained a set of subtyping results *R_i,c,k,σ_*, where *c* = 1,2,⋯,11 is the index of the data combination used, *k* = 2,3,⋯,8 is the number of subtypes to calculate, and *σ* = 0,0.5,1,2,3,4 is the noise level. For each method *i*, we calculated the NMI and ARI between the result *R*_*i,c,k*,0_ (the result of using original data) and the result *R_i,c,k,σ′_*(*σ*′ = 0.5,1,2,3,4) obtained when different levels of noise are introduced into the data. As PINS could not cluster samples into a specific number of clusters, we calculated the NMI and ARI between *R*_*i,c*,0_ and *R_i,c,σ′_*(*σ*′ = 0.5,1,2,3,4). The calculations of robustness were reported in S4 File.

**Fig 7:**
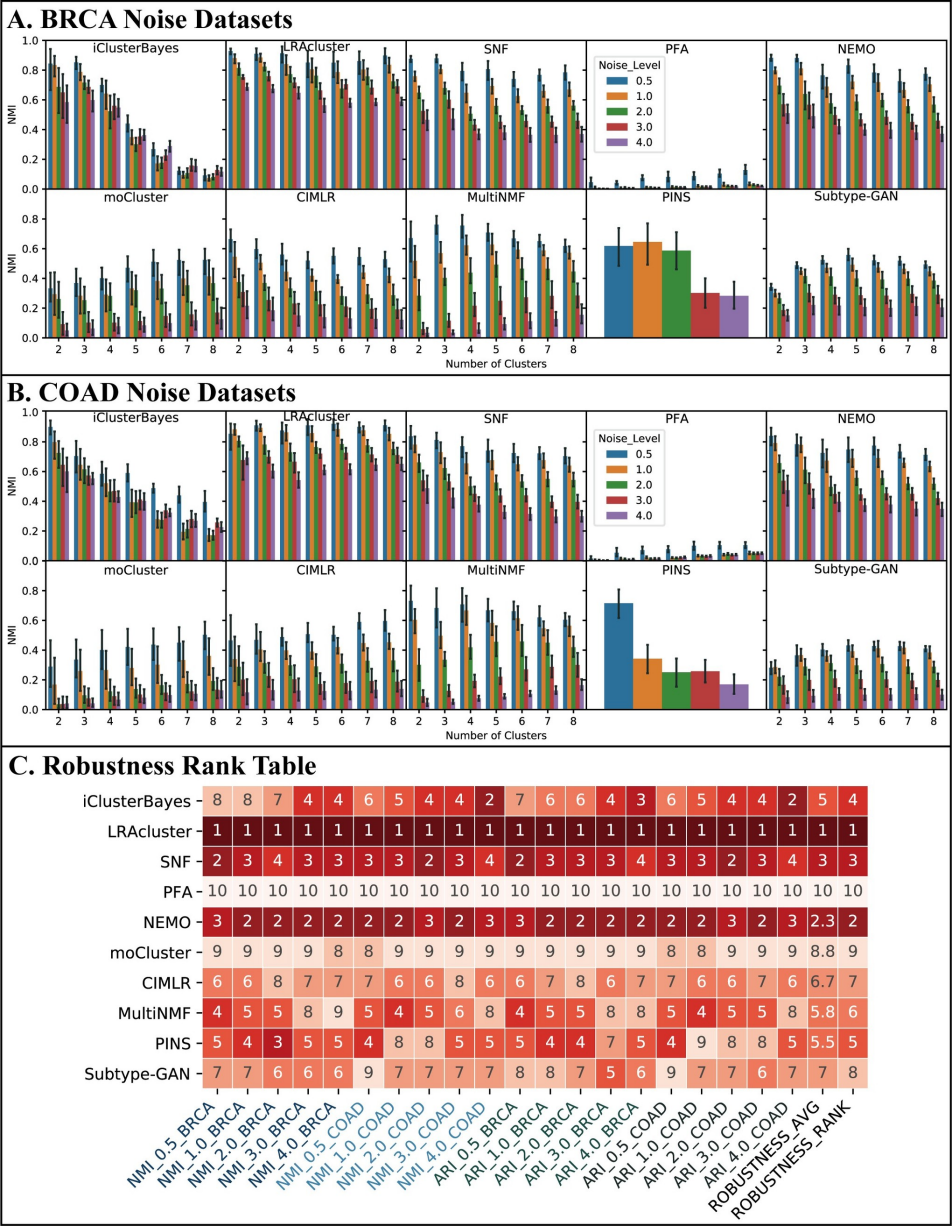
Robustness performance. A robust method should satisfy two criteria: when the number of clusters is fixed, its NMI decreases slowly as the noise level increases; for a fixed level of noise, the NMI has little difference among the different number of clusters. We show the NMI comparisons of (A) BRCA noise datasets and (B) COAD noise datasets. The x-coordinate of figures is the number of clusters on which there are five bars in each cluster number. Each bar represents the average NMI over all 11 combinations at the current noise level datasets on the given number of clusters. The confidence interval around the average NMI is plotted using an error bar. (C) Robustness rank table. The rank items listed under the table include the information of metrics, noise level, and cancer which are connected by underlines.

Using the BRCA noise datasets, we found that LRAcluster is the most robust method since it has the largest average NMI at all noise levels (Fig 7A). When the number of clusters is fixed, its NMI decreases slowly as the noise level increases. For a fixed level of noise, the NMI of LRAcluster has little difference among the different number of clusters. The robustness of NEMO and SNF are similar to LRAcluster, but their NMI are lower than LRAcluster’s under the same condition and decrease more quickly with the noise level. The robustness of iClusterBayes is good at *k* = 2,3,4, and even better than SNF when *k* = 2,3. However, when *k*>4, the NMI decreases sharply and becomes much lower than the NMI of SNF under the same condition. MultiNMF has the acceptable robustness performance at only *k* = 2,3. Fig 7B shows the results on the COAD noise datasets, from which similar observations can be made. From the results of ARI, we had similar observations.

Fig 7C shows the rank table of robustness comparison. LRAcluster is ranked in the first place in all 20 tests and is thus the most robust method, followed by NEMO and SNF.

### Computational efficiency

The computation time required to complete the tasks is an important factor to consider when practitioners choose among different integration methods, especially when the amount of data to be processed is huge. Sometimes, a user may prefer a less accurate method over a method that takes a long time to finish.

Dataset group #1 and the Pan-cancer dataset in Dataset group #3 were used to evaluate the computational efficiency of the integration methods. The scales of the datasets used in the evaluation are listed in Table 4. In our experiments, we used the default parameter settings in the R package/MATLAB source code of these methods. To obtain the best results of LRAcluster, we tried the dimension parameter from 1 to 10 and the sum of the running time these 10 trials took was evaluated. As one of the essential steps of moCluster is to determine the number of latent variables using permutation and elbow test, we also tried this parameter from 1 to 10. The running time of the K-means clustering algorithm was not included for LRAcluster, PFA, and MultiNMF because these methods derived the matrices after dimension reduction instead of the clustering results, and the running time of K-means had little influence on the comparisons and analysis.

**Table 4:** Data scales of different datasets.

Dataset	Cancer	Sample	mRNA	miRNA	Methylation	CNV
Complete dataset of Dataset group #1	BRCA	759	18206	368	19049	19568
	COAD	291	17261	375	19052	19551
	KIRC	314	18465	352	19056	19552
	LUAD	450	18310	427	19052	19551
	LUSC	363	18599	423	19060	19551
	ACC	77	18034	845	18711	19551
	KIRP	273	18241	769	18715	19551
	LIHC	364	17946	846	18714	19551
	THYM	119	18354	1018	18716	19551
Significant dataset of Dataset group #1	BRCA	759	2000	200	2000	1974
	COAD	291	2000	200	2000	1449
	KIRC	314	2000	200	2000	2102
	LUAD	450	2000	200	2000	3446
	LUSC	363	2000	200	2000	3074
	ACC	77	2000	200	2000	524
	KIRP	273	2000	200	2000	1023
	LIHC	364	2000	200	2000	2050
	THYM	119	2000	200	2000	991
Pan-cancer dataset of Dataset group #3	-	2177	16636	315	19048	19551

For each method and each combination of data types, the total running time is the sum of the time the method takes to complete the computation for all the settings for the number of clusters from 2 through 8. The calculations of computational efficiency were reported in S5 File.

**Fig 8:**
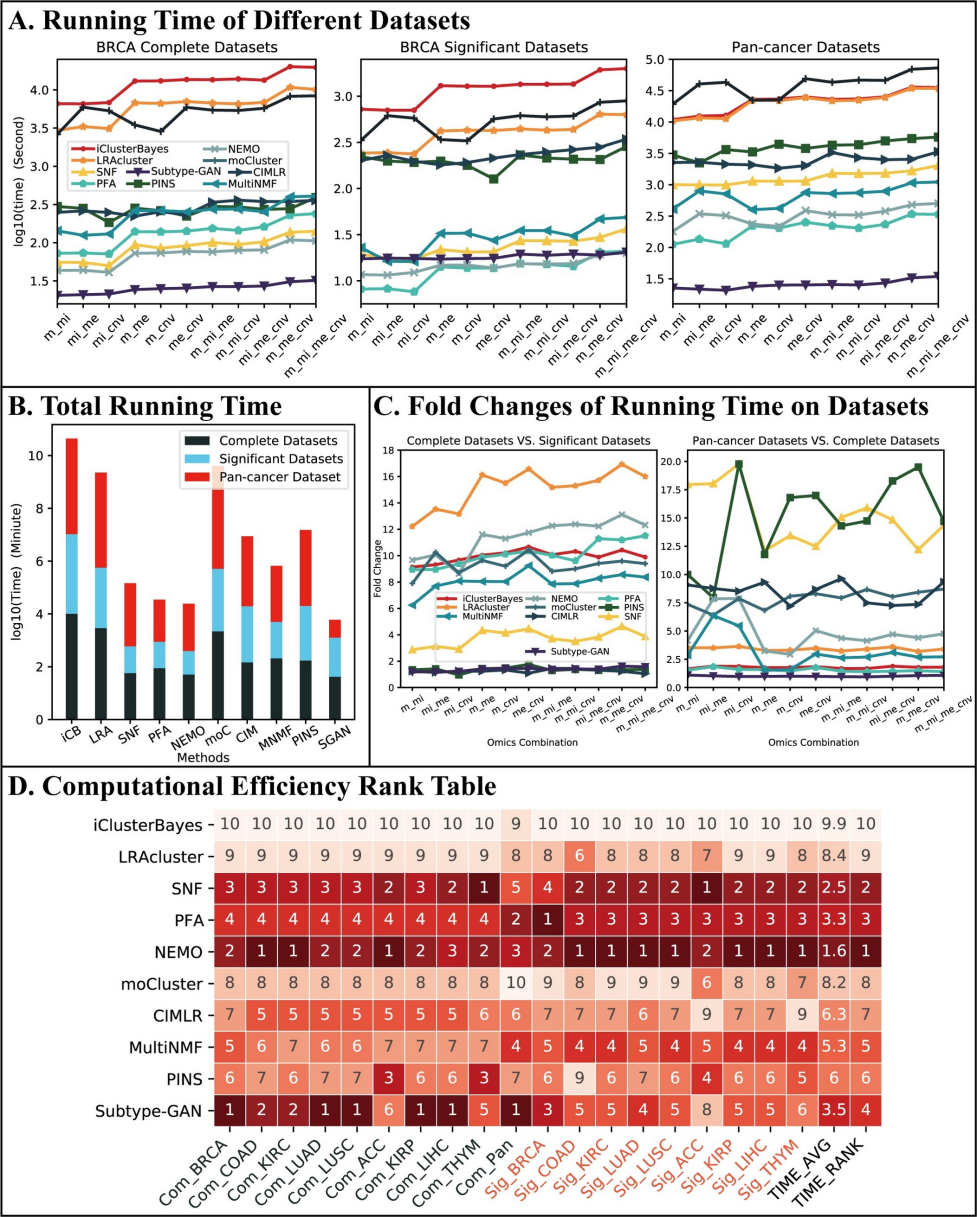
Computational efficiency performance. (A) Running time of different datasets. Here, we only included the results for the BRCA datasets in Dataset #1 and the Pan-cancer datasets in Dataset #3. The difference between the running times of these methods is similar to other cancer datasets but less significant as those datasets are much smaller than BRCA datasets. The data combinations are placed along the x-coordinate in ascending order of the number of participating data types and data scales. (B) Total running time. (C) Fold changes of running time on different datasets. (D) Computational efficiency rank table. The rank items listed under the table include the datasets and cancer which are connected by underlines. “Com” and “Sig” represent complete and significant datasets, respectively.

Fig 8A shows the comparison of the running time. Due to the large difference in running time of different methods, we used the log 10 transformation of running time for illustration. It is clear that the three statistics-based methods, i.e. iClusterBayes, LRAcluster and moCluster, cost too much more time than the network/graph-based methods. With the increase of data scale, the running time of all the methods increases. The comparison of the total running time, including running times on all combinations and all datasets, is shown in Fig 8B from which the same conclusion can be drawn. The deep learning-based method, Subtype-GAN, cost lowest time in complete and Pan-cancer datasets so that it performed the best in total running time.

The scale of a dataset is decided by two factors: the number of samples and the number of features per sample. To get a better picture about the computational efficiency of the integration methods, we analyzed the influence of the two factors on the running time. Fig 8C shows the fold change (i.e., ratio) of the running time on the complete BRCA datasets to the running time on significant BRCA datasets and the fold change of the running time on the Pan-cancer datasets to the running time on the complete BRCA datasets. From Table 4, we see that the significant datasets of Dataset group #1 all have the same number of samples as the complete datasets but have much fewer features, while the Pan-cancer datasets in Dataset group #3 have almost the same number of features as the complete datasets of Dataset group #1, but have much more samples. These, together with the results in Fig 8C, indicate that for statistics-based methods, such as LRAcluster, an increase in the number of features has a much more severe impact on their computational efficiency than an increase in the number of features. On the other hand, for network-based methods such as SNF and CIMLR, the number of samples is a more significant factor in their computational efficiency. The running time of the deep learning-based method Subtype-GAN is hardly affected by the increase in samples and features which has a significant advantage in large-scale datasets.

Fig 8D shows the running-time-based rank table of these methods. NEMO and SNF ranked first and second, respectively, and are the most efficient methods. We note that PFA, while being ranked in the third place, takes a little amount of total time than SNF (Fig 8B). This is because its running time on the Pan-cancer datasets is much lower than SNF. For Subtype-GAN which ranked fourth, although it runs fastest in both complete and Pan-cancer datasets that have a large number of features, it costs much more time than network-based methods in significant datasets.

### Influences of different omics data and their integration on cancer subtyping

Using our experiment results on the accuracy of the integration methods over various data combinations and cancers, we explored the influence of different omics data and their combinations on the effectiveness of data integration for cancer subtyping. We focused on two issues. The first one is whether a specific type of omics data has similar discriminative power for subtyping on different cancers. The second one is whether there are combinations of data types that are effective for the majority of the integration methods and cancers.

To quantify the relative influence of different types of omics data on the effectiveness of data integration for a specific cancer, we use the Weighted Average Z-Score (among the group of 4 omics data types) that takes into consideration the accuracy measures from our experiments on the integration methods under all accuracy metrics, including precision, NMI, ARI, F-measure, silhouette coefficient and transformed log-rank test p-value. We notice that the strategies of suggesting *k* in these ten methods are different. The performance based on the method-suggested *k* and all possible *k* are inconsistent for some methods. There could be two possible reasons: (1) the procedure of suggesting *k* in the methods may not work for our constructed benchmarking datasets; (2) the performance based on the method-suggested *k* are not optimal. Therefore, in order to avoid the bias introduced by the selection of *k*, we do not take the results using the method-suggested *k* into consideration. Moreover, as PINS cannot generate the subtyping results for a specific *k*, we do not consider the results of PINS in the following analysis.

For a given cancer, the Weighted Average Z-Scores are calculated as follows. For each type of omics data and each integration method, we calculate the average accuracy measure (over all the accuracy metrics) of the integration method applied to a specific data combination that contains this type of omics data. Since each omics data type belongs to exactly 7 of the 11 possible combinations, there are 7 measurements for each accuracy measure, and we take the mean of these 7 values as the Overall Accuracy Measure of the omics data type on a specific metric. We then calculate the z-scores (with respect to the particular integration method) among the four types of omics data, using their Overall Accuracy Measures. The weighted average is defined in a similar way as the weighted rank in the Section “Accuracy”. The Weighted Average Z-Score of a type of omics data is its average z-scores over all the integration methods. Regarding the use of different metrics, we mention that, as we had calculated the silhouette coefficient and the p-value of these methods under different settings for the number of clusters (from 2 to 8), we took the average of them. For the other four metrics, we used the value obtained in the experiments directly. When we evaluated the clinical significance of KIRP, we found that many zero p-values occurred which was abnormal, and could not do the log transformation. Therefore, we did not analyze KIRP in this work.

**Fig 9:**
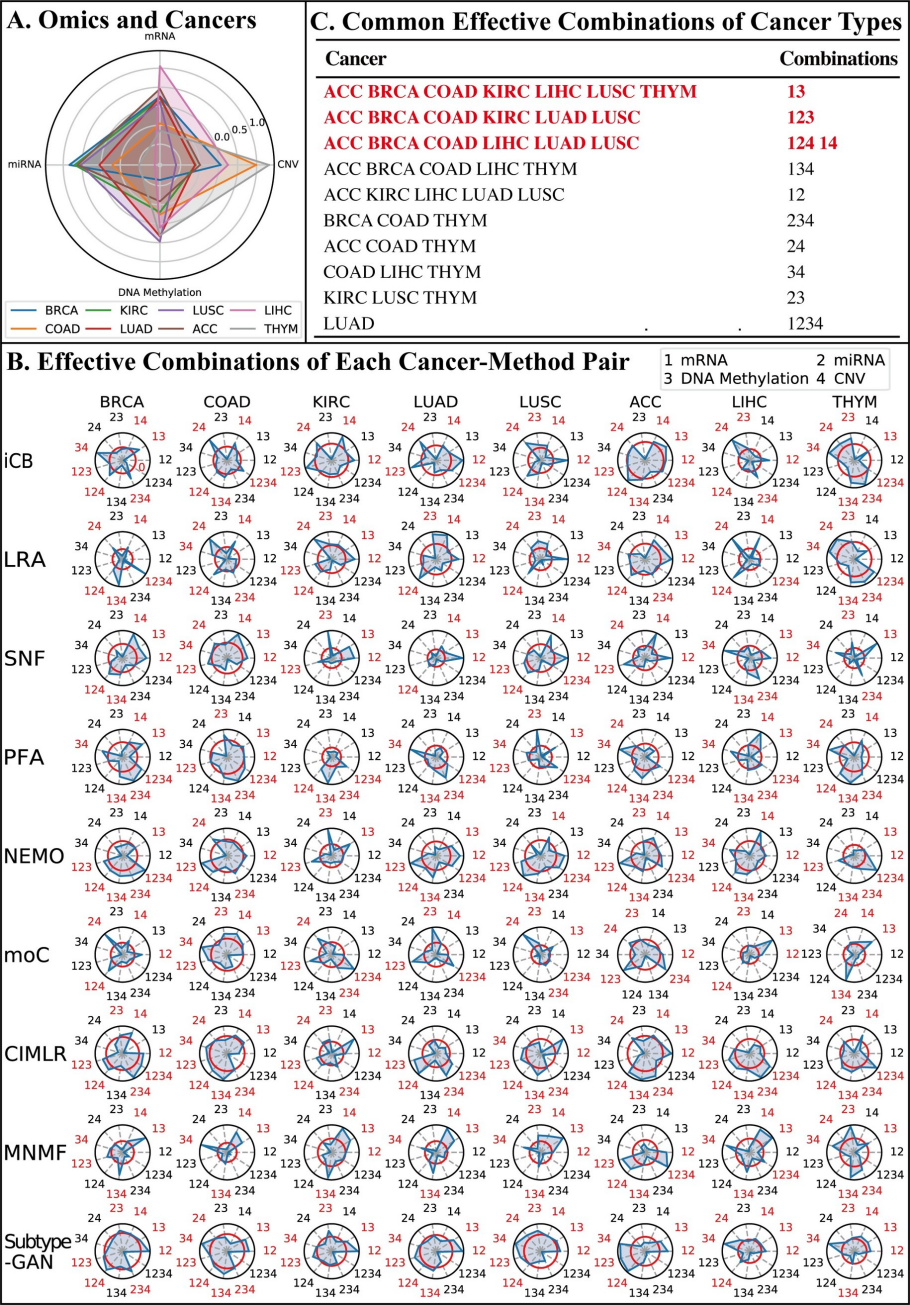
Influences of different omics data and their integration on cancer subtyping. (A) Influence of different omic types to different cancers on cancer subtyping. On the radar plot, each quadrilateral represents a cancer type and each vertex of the quadrilateral represents the influence (i.e. Weighted Average Z-score) of a type of omic data with regard to that cancer. (B) Effective combinations of each cancer-method pair. Each vertex in the radar plots represents the weighted average z-score of a specific data combination with respect to a particular pair of cancer and method. In the plots, we use integers 1, 2, 3, and 4 to label the four omics data types mRNA expression, miRNA expression, DNA methylation, and CNV, respectively, so that a data combination can be written as a sequence of digits. For example, the sequence “134” corresponds to the combination of the three data types: mRNA expression, DNA methylation, and CNV. The red circle on each radar plot represents the z-score of zero. Combinations with a positive average weighted z-score are colored red and are considered to be effective for that cancer-method pair. (C) Common effective combinations of cancer types. The effective combinations for most cancer types are colored red.

Fig 9A summarizes the results. We see that LIHC are mainly affected by the mRNA expression, BRCA, KIRC, LUSC, and ACC are mainly affected by the miRNA expression, COAD and THYM are mainly affected by CNV, and LUAD is affected mainly by DNA methylation. This observation confirms that the effectiveness of data integration on subtyping of different cancers might be affected by different omics data. An even more interesting observation is that the effectiveness of data integration on the subtyping of the two lung cancers LUAD and LUSC is largely influenced by two different types of omics data. This might help shed light on the origin, driver, or causal events of lung cancers.

To analyze the effectiveness of different data combinations, we calculated the weighted average z-scores among all possible combinations of data types for each pair of cancer and the integration method. For a given cancer and integration method, the weighted average z-scores are based on the accuracy measures of the method applied on the datasets of that cancer using different data combinations, and the strategies of choosing measurements and the calculation of weighted average z-scores are the same as mentioned above. The radar plots of these weighted average z-scores are shown in Fig 9B, and we use integers 1, 2, 3, and 4 to label the four omics data types mRNA expression, miRNA expression, DNA methylation, and CNV, respectively, so that a data combination can be written as a sequence of digits.

As these radar plots show, we find that there are no combinations in any cancer datasets that are effective for all nine methods. The situation becomes better when we consider combinations that are effective for five or more methods. For example, for the BRCA datasets, the collection of data combinations {“13”, “14”, “123”, “124”, “134”, and “234”} are all effective for at least five integration methods, and we consider these combinations as effective combinations of BRCA. In the following analysis, we summarize the effective combinations of each cancer to find the combinations which are effective for most cancers. It turns out that the combination “13” (i.e. mRNA expression and DNA methylation) is effective for seven cancers. The combinations “14” (i.e. mRNA expression and CNV), “123” (i.e. mRNA expression, miRNA expression, and DNA methylation), “124” (i.e. mRNA expression, miRNA expression, and CNV) are effective for six cancers.

To further verify the effectiveness of the combinations identified in the above analysis, we compared the performance of the integration methods using these combinations with the performance of three widely-used clustering methods (K-means, spectral clustering, and hierarchical clustering) applied to individual omics data types. We used the silhouette coefficients and the transformed p-values calculated in the log-rank test for the comparison.

**Fig 10:**
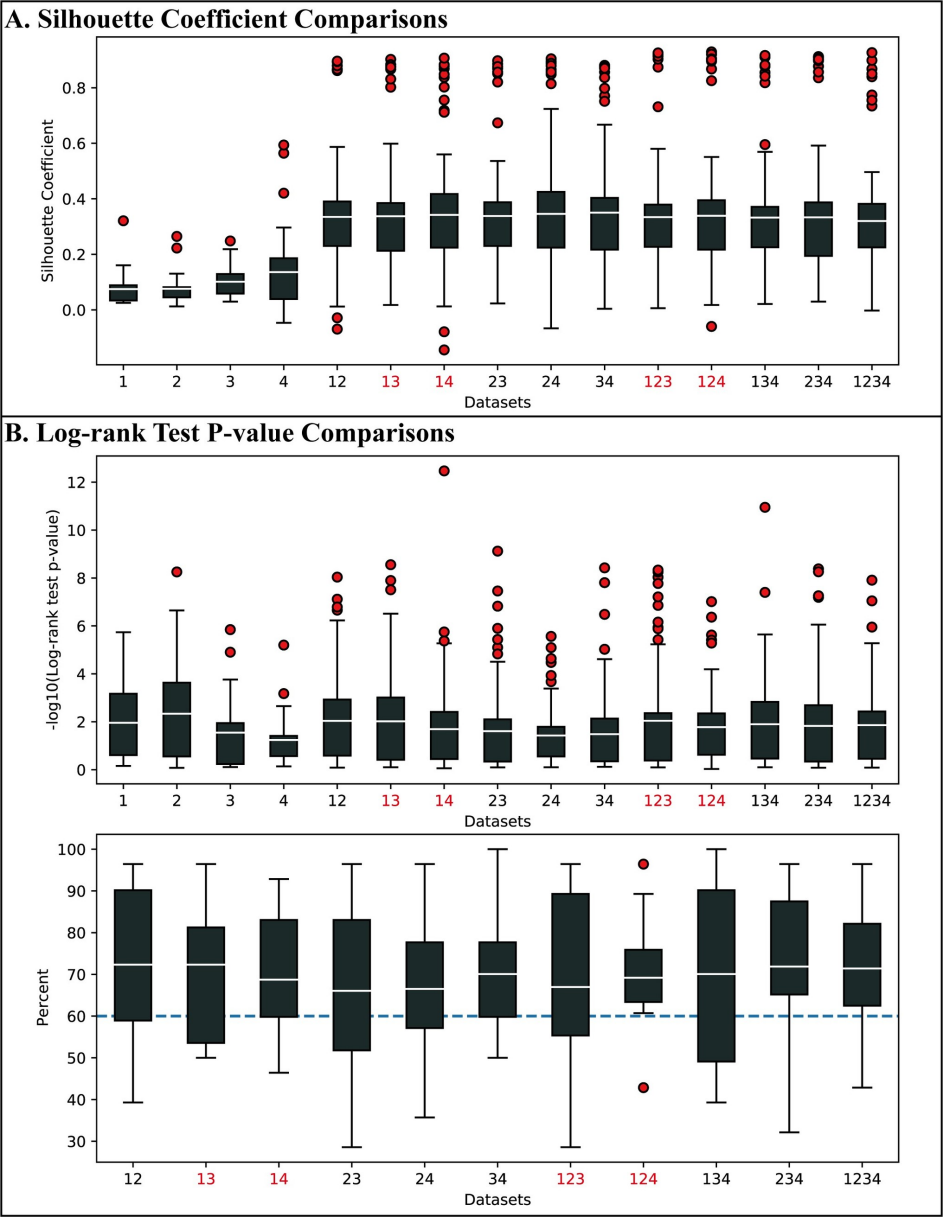
Comparison of the performance of the integration methods using effective combinations with the performance of clustering methods applied to individual omics data types. (A) Silhouette coefficients comparison. (B) Transformed p-values of the log-rank test comparison. In the bottom plot, each point in the box is the improvement percentage for a specific cancer type on that combination.

Because of the characteristic of multi-omic data, high dimensions relative to the small samples, most of the existing methods can not properly separate the samples in the original space. For achieving better clustering results in multi-omics datasets integration, many effective integration methods project features of each omic data into a new integrated space and then cluster samples in this integrated space. In order to show the advantages of data integration, we used the silhouette coefficient results based on integrated space to do the comparison. As Fig 10A shows, the average silhouette coefficient achieved by the nine integration methods over all parameter settings (number of clustering 2 to 8) on each of the 11 possible combinations is consistently higher than the average silhouette coefficient achieved by the three clustering methods using individual data types over the same range of parameter setting, with performance gain of the effective combinations identified in the preceding analysis being much more significant. The comparison of the log-transformed p-values of the log-rank test is summarized in the top plot in Fig 10B. “123”, “13” ranked top four of all 15 items while “124” and “14” ranked the ninth and tenth. In addition, the results of integration methods have more outliers (depicted in red circles), suggesting that multi-omics integration could potentially find subtypes with more significant differences in survival analysis.

As there is no gold standard, a well-known challenging task to use unsupervised clustering methods is the selection of the parameter setting for the number of clusters. To further understand the power and clinical advantage of data integration in cancer subtyping, we compared the p-value of single-omic clustering-based subtyping methods and integration methods when they use the same (and fixed) number of clusters *k*. For each data combination and each cancer, we calculated the average p-value of the eight integration methods and the average p-value of three single-omic clustering methods, using the same number of clusters *k*. We then counted the total number of cases (over the possible values of *k* = 2 to 8) where integration methods using the data combination have improved performance (i.e. a greater transformed p-value) over the single-omic-based clustering methods. The bottom plot in Fig 10B shows the percentages of improvements of using each combination. The average values of all eleven combinations are over 60%. These observations indicate that the improvement of using effective combinations in integration methods over single types of omic-data is not only significant but also robust in a certain degree over the choice of the method’s parameter.

## Discussion

Many multi-omics integration methods have been proposed to improve our understanding of cancer. Cancer subtyping can be used in precision medicine to help patients receive more accurate and personalized treatments based on their response to different therapies and drugs. In this study, we have conducted a comprehensive evaluation and comparison of ten representative multi-omics integration methods in terms of their accuracy, robustness, and computational efficiency. Using our benchmarking datasets constructed from collections of real cancer data, we were able to evaluate the accuracy of these methods from the clinic perspective as well as the computational perspective. From our experiments and analyses, we observed that the methods NEMO and SNF perform very well in all three criteria, i.e. accuracy, robustness, and computation efficiency, which is mainly because the strategies that they adopt can capture both shared and specific information from different omics data and make the integrated similarity networks retain more information from every single similarity network with low-level noise. The other methods have certain limitations. For example, LRAcluster is the best in robustness, but has poor computational efficiency; iClusterBayes, while being good in accuracy and robustness, has the worst computational efficiency; PFA has the poor robustness and accuracy, but has better performance in running time. Among the seven methods that can suggest an optimal *k* after specifying a *k*-max, we must point out that PINS is the most friendly-used method because its automatic procedure of estimating *k* does not require users to do any additional work. In contrast, some methods (e.g. iClusterBayes and moCluster) require users to select *k* based on their generated curves, however, it could be a tough task for users because the breakpoint of the curve cannot be easily determined or inconsistent *k* could be recommended by two different types of plots. In summary, NEMO and SNF are recommended for general cancer subtyping tasks, however, users may also want to consider the other eight methods depending on their specific purpose or under certain circumstances.

Using our results on the accuracy, we have also analyzed the influence of different omics data types and their combinations on cancer subtyping. Our analysis shows that the influence of these omics data types varies, and several commonly-used combinations of omics data types can indeed improve the accuracy of all the ten methods as measured by both clustering and clinical metrics. On the other hand, our analysis indicates that integrating more types of omics data may negatively impact the performance on cancer subtyping, refuting the widely held intuition that incorporating more types of omics data always helps produce better results [113]. From our experiments, it is clear that the results obtained by using the four omics data types for integration analysis were not always better than the results obtained by using three or two-omics data types. Similarly, the results of the three-omics integration were not always better than the results of two-omics integration. While this observation is counter-intuitive and deserves further investigation, we believe that it is the consequence of three intertwined factors: (1) the negatively-correlated noises in the omics data which may cancel out useful information; (2) the redundancy in different types of omics data; and (3) the computational/statistical challenges that integrating excessive datasets impose on the integration methods preventing them from making the best use of the information, if any, in the multi-omics datasets, to calculate optimal solutions.

It is worthwhile to mention another surprising observation on the significance of DNA methylation data in the effectiveness of integration methods. Ramazzotti et al. proposed their method CIMLR [13] based on multiple kernel learning and employed CIMLR to integrate point mutation, CNA, DNA methylation, and gene expression four omics data for cancer subtyping. They found that in this four-omic combination, each of gene expression and methylation accounted for 30–50% of the kernel weight in most of 36 cancers. Our analysis on the effectiveness of the 11 possible combinations, however, shows that only one combination in 2 and 3 omics datasets can be considered to be effective. This is unexpected as methylation had been proven to play an important role in cancer and has been the most common data type used in previous research on integrated multi-omics data for cancer subtyping. We leave it to future work to understand this unexpected phenomenon, but speculate that it is the result of the following three factors of our experiments: (1) certain characteristics of the methylation data that do not fit the model assumption of some of the ten integration methods, resulting in a much lower overall effectiveness score for those of the methylation-participating combinations in our evaluation; (2) the data processing step in which we mapped all the features to genes in methylation and CNV datasets as previous studies did, resulting in a loss of some significant information; and (3) performance criterion that uses clinical-based metrics as well as clustering-based metrics, in comparison to previous studies where only one metric may be used.

As more types of omics data have being generated for cancer patients and more integration methods, especially deep learning-based methods, have being proposed, new data types such as proteomics and single-cell omics data as well as machine learning-based methods should be considered in the future development of the benchmarking framework for data integration.

## Supporting information

S1 File Supplementary File. Evaluation and comparison of multi-omics data integration methods for cancer subtyping. **S1 Fig. Silhouette coefficient comparison based on integrated space of Dataset group #1 Nine-cancer Datasets.** We use “iCB”, “LRA”, “moC”, “CIM”, “MNMF” and “SGAN” to represent iClusterBayes, LRAcluster, moCluster, CIMLR, MultiNMF, and Subtype-GAN, respectively. (A) Silhouette coefficient based on the suggested k of methods. We set k-max as 8 and let each method suggest the best k. Each of the 11 data points in a box represents a silhouette coefficient of the subtyping results based on the method suggested k obtained by the corresponding method using one of the 11 possible combinations of data types. (B) Silhouette coefficient based on all the possible k. Each of the 11 data points in a box represents the average silhouette coefficient of the subtyping results from k = 2 to 8 obtained by the corresponding method using one of the 11 possible combinations of data types. **S1 Table. Suggested *k* of each method using Dataset group #1 Nine-cancer Datasets.** Notations: B-BRCA, C-COAD, KC-KIRC, LA-LUAD, LS-LUSC, A-ACC, KP-KIRP, LI-LIHC, T-THYM. m-mRNA expression, mi-miRNA expression, me-DNA methylation, cnv-copy number variation. **S2 Table. Suggested *k* of each method using Dataset group #3 Gold Standard Datasets.** Notations: m-mRNA expression, mi-miRNA expression, me-DNA methylation, cnv-copy number variation.
(DOCX)

S2 File Accuracy Calculation of Dataset group #1. (XLSX)

S3 File Accuracy Calculation of Dataset group #3. (XLSX)

S4 File Robustness Calculation of Dataset group #2. (XLSX)

S5 File Computational Efficiency Calculation. (XLSX)

S6 File Selected samples and features. (XLSX)
