# Supplementary material for: Evaluation and comparison of multi-omics data integration methods for cancer subtyping
Source: PLoS Comput Biol. 2021 Aug 12;17(8):e1009224. doi: 10.1371/journal.pcbi.1009224 (PMC8384175; doi:10.1371/journal.pcbi.1009224)
Supplement: S1 File — Evaluation and comparison of multi-omics data integration methods for cancer subtyping. S1 Fig. Silhouette coefficient comparison based on integrated space of Dataset group #1 Nine-cancer Datasets. We use “iCB”, “LRA”, “moC”, “CIM”, “MNMF” and “SGAN” to represent iClusterBayes, LRAcluster, moCluster, CIMLR, MultiNMF, and Subtype-GAN, respectively. (A) Silhouette coefficient based on the suggested k of methods. We set k-max as 8 and let each method suggest the best k. Each of the 11 data points in a box represents a silhouette coefficient of the subtyping results based on the method suggested k obtained by the corresponding method using one of the 11 possible combinations of data types. (B) Silhouette coefficient based on all the possible k. Each of the 11 data points in a box represents the average silhouette coefficient of the subtyping results from k = 2 to 8 obtained by the corresponding method using one of the 11 possible combinations of data types. S1 Table. Suggested k of each method using Dataset group #1 Nine-cancer Datasets. Notations: B-BRCA, C-COAD, KC-KIRC, LA-LUAD, LS-LUSC, A-ACC, KP-KIRP, LI-LIHC, T-THYM. m-mRNA expression, mi-miRNA expression, me-DNA methylation, cnv-copy number variation. S2 Table. Suggested k of each method using Dataset group #3 Gold Standard Datasets. Notations: m-mRNA expression, mi-miRNA expression, me-DNA methylation, cnv-copy number variation. (DOCX) [file pcbi.1009224.s001.docx]

**Supplementary File**

**Evaluation and comparison of multi-omics data integration methods for cancer subtyping**

Ran Duan^1^, Lin Gao^1*^, Yong Gao^2^, Yuxuan Hu^1^, Han Xu^1^, Mingfeng Huang^1^, Kuo Song^1^, Hongda Wang^1^, Yongqiang Dong^1^, Chaoqun Jiang^1^, Chenxing Zhang^1^, Songwei Jia^1^

^1^ School of Computer Science and Technology, Xidian University, Xi’an, China

^2^ Department of Computer Science, The University of British Columbia Okanagan, Kelowna, British Columbia, Canada

**Recommended *k* of each method**

In this work, we chose ten typical methods to evaluate, compare, and analyze, including iClusterBayes, LRAcluster, SNF, PFA, NEMO, moCluster, CIMLR, MultiNMF, PINS and Subtype-GAN. We notice that, in these methods, iClusterBayes, SNF, PINS, NEMO, moCluster, CIMLR and Subtype-GAN have their criteria to estimate the best number of clusters based on different strategies. According to the criteria of these methods, we set the *k*-max as 8 and ran these methods using our Dataset group #1 Nine-cancer datasets and Dataset group #3 Gold Standard Datasets. S1 and S2 Tables show every suggested *k* of different methods.

**S1 Table. Suggested *k* of each method using Dataset group #1 Nine-cancer Datasets.**

| **Methods** | **Data**  **Combination** | **Complete Datasets** | | | | | | | | | **Significant Datasets** | | | | | | | | |
| --- | --- | --- | --- | --- | --- | --- | --- | --- | --- | --- | --- | --- | --- | --- | --- | --- | --- | --- | --- |
|  |  | **B** | **C** | **KC** | **LA** | **LS** | **A** | **KP** | **LI** | **T** | **B** | **C** | **KC** | **LA** | **LS** | **A** | **KP** | **LI** | **T** |
| **iCluster Bayes** | **m_mi** | **4** | **3** | **3** | **4** | **3** | **2** | **2** | **2** | **2** | **3** | **2** | **2** | **2** | **3** | **2** | **2** | **2** | **2** |
|  | **m_me** | **4** | **3** | **4** | **4** | **3** | **2** | **2** | **2** | **2** | **3** | **2** | **3** | **3** | **3** | **2** | **3** | **3** | **2** |
|  | **m_cnv** | **4** | **3** | **3** | **4** | **3** | **2** | **4** | **3** | **2** | **3** | **3** | **2** | **4** | **2** | **2** | **3** | **4** | **2** |
|  | **mi_me** | **2** | **3** | **4** | **2** | **2** | **2** | **2** | **2** | **2** | **2** | **2** | **3** | **2** | **2** | **2** | **2** | **2** | **2** |
|  | **mi_cnv** | **3** | **3** | **3** | **4** | **3** | **2** | **3** | **4** | **2** | **3** | **2** | **2** | **3** | **2** | **3** | **2** | **2** | **2** |
|  | **me_cnv** | **3** | **2** | **4** | **2** | **3** | **2** | **3** | **3** | **2** | **4** | **3** | **3** | **3** | **2** | **2** | **3** | **2** | **2** |
|  | **m_mi_me** | **4** | **3** | **3** | **3** | **3** | **2** | **4** | **4** | **2** | **3** | **2** | **3** | **3** | **3** | **2** | **2** | **4** | **2** |
|  | **m_mi_cnv** | **4** | **4** | **3** | **4** | **3** | **2** | **3** | **4** | **3** | **3** | **3** | **3** | **4** | **2** | **2** | **2** | **3** | **2** |
|  | **m_me_cnv** | **4** | **3** | **4** | **4** | **4** | **2** | **3** | **4** | **2** | **3** | **2** | **3** | **3** | **2** | **2** | **3** | **4** | **2** |
|  | **mi_me_cnv** | **4** | **2** | **4** | **2** | **3** | **2** | **3** | **3** | **2** | **4** | **2** | **2** | **3** | **2** | **2** | **3** | **2** | **2** |
|  | **m_mi_me_cnv** | **4** | **3** | **4** | **4** | **3** | **2** | **3** | **3** | **2** | **3** | **2** | **3** | **4** | **2** | **2** | **2** | **4** | **2** |
| **SNF** | **m_mi** | **2** | **2** | **3** | **2** | **2** | **2** | **3** | **2** | **3** | **2** | **3** | **2** | **2** | **2** | **2** | **3** | **2** | **2** |
|  | **m_me** | **2** | **3** | **4** | **2** | **3** | **2** | **3** | **3** | **3** | **2** | **3** | **4** | **2** | **2** | **2** | **3** | **2** | **2** |
|  | **m_cnv** | **2** | **2** | **4** | **2** | **2** | **3** | **5** | **2** | **2** | **3** | **2** | **4** | **4** | **3** | **3** | **6** | **2** | **2** |
|  | **mi_me** | **2** | **2** | **4** | **2** | **2** | **2** | **3** | **5** | **3** | **3** | **3** | **6** | **2** | **2** | **2** | **2** | **4** | **2** |
|  | **mi_cnv** | **2** | **2** | **4** | **2** | **2** | **3** | **3** | **2** | **2** | **3** | **2** | **3** | **2** | **2** | **2** | **8** | **2** | **2** |
|  | **me_cnv** | **4** | **2** | **3** | **2** | **2** | **3** | **2** | **3** | **2** | **3** | **2** | **4** | **2** | **2** | **3** | **2** | **2** | **2** |
|  | **m_mi_me** | **2** | **3** | **3** | **2** | **2** | **2** | **3** | **2** | **3** | **3** | **3** | **2** | **2** | **2** | **2** | **3** | **2** | **2** |
|  | **m_mi_cnv** | **2** | **2** | **4** | **2** | **2** | **3** | **2** | **2** | **2** | **2** | **2** | **3** | **2** | **2** | **2** | **3** | **4** | **3** |
|  | **m_me_cnv** | **3** | **2** | **3** | **2** | **2** | **3** | **2** | **3** | **3** | **3** | **2** | **3** | **2** | **2** | **3** | **2** | **2** | **2** |
|  | **mi_me_cnv** | **3** | **2** | **3** | **2** | **2** | **3** | **2** | **3** | **3** | **3** | **2** | **3** | **2** | **2** | **2** | **2** | **2** | **2** |
|  | **m_mi_me_cnv** | **2** | **2** | **3** | **2** | **2** | **3** | **2** | **2** | **3** | **3** | **2** | **3** | **2** | **2** | **2** | **4** | **2** | **2** |
| **PINS** | **m_mi** | **7** | **18** | **8** | **39** | **16** | **4** | **10** | **12** | **5** | **14** | **14** | **25** | **14** | **22** | **4** | **22** | **28** | **8** |
|  | **m_me** | **11** | **5** | **11** | **34** | **32** | **8** | **4** | **11** | **8** | **51** | **30** | **2** | **19** | **21** | **2** | **2** | **3** | **7** |
|  | **m_cnv** | **14** | **2** | **2** | **11** | **11** | **7** | **2** | **2** | **5** | **26** | **3** | **8** | **27** | **23** | **3** | **3** | **16** | **2** |
|  | **mi_me** | **9** | **3** | **2** | **24** | **11** | **2** | **3** | **17** | **13** | **32** | **18** | **23** | **17** | **15** | **4** | **2** | **14** | **7** |
|  | **mi_cnv** | **9** | **14** | **6** | **15** | **11** | **10** | **2** | **14** | **6** | **7** | **4** | **4** | **15** | **17** | **6** | **2** | **25** | **4** |
|  | **me_cnv** | **4** | **2** | **2** | **13** | **3** | **4** | **5** | **30** | **3** | **2** | **4** | **4** | **16** | **19** | **6** | **2** | **24** | **5** |
|  | **m_mi_me** | **8** | **2** | **2** | **2** | **2** | **3** | **2** | **4** | **3** | **2** | **7** | **2** | **4** | **3** | **3** | **2** | **2** | **9** |
|  | **m_mi_cnv** | **8** | **2** | **3** | **9** | **2** | **2** | **2** | **2** | **2** | **2** | **3** | **2** | **4** | **7** | **2** | **2** | **3** | **2** |
|  | **m_me_cnv** | **4** | **2** | **2** | **7** | **8** | **3** | **3** | **5** | **3** | **3** | **3** | **2** | **4** | **3** | **3** | **2** | **2** | **2** |
|  | **mi_me_cnv** | **7** | **2** | **2** | **4** | **3** | **2** | **3** | **3** | **4** | **2** | **3** | **2** | **5** | **3** | **2** | **3** | **2** | **2** |
|  | **m_mi_me_cnv** | **8** | **5** | **4** | **4** | **5** | **3** | **4** | **7** | **3** | **5** | **7** | **2** | **2** | **5** | **3** | **3** | **2** | **2** |
| **NEMO** | **m_mi** | **7** | **2** | **6** | **7** | **4** | **6** | **8** | **8** | **7** | **5** | **2** | **7** | **7** | **3** | **5** | **3** | **8** | **2** |
|  | **m_me** | **3** | **2** | **4** | **4** | **4** | **4** | **3** | **8** | **3** | **3** | **2** | **3** | **4** | **3** | **7** | **3** | **5** | **2** |
|  | **m_cnv** | **3** | **2** | **4** | **6** | **2** | **3** | **8** | **3** | **5** | **3** | **2** | **3** | **4** | **2** | **6** | **7** | **6** | **7** |
|  | **mi_me** | **2** | **3** | **5** | **5** | **6** | **2** | **3** | **4** | **3** | **3** | **3** | **5** | **5** | **3** | **4** | **8** | **8** | **2** |
|  | **mi_cnv** | **7** | **2** | **6** | **5** | **2** | **6** | **7** | **7** | **5** | **3** | **3** | **4** | **5** | **2** | **8** | **7** | **3** | **6** |
|  | **me_cnv** | **3** | **7** | **6** | **6** | **5** | **6** | **2** | **4** | **3** | **3** | **3** | **5** | **6** | **2** | **8** | **8** | **4** | **6** |
|  | **m_mi_me** | **3** | **3** | **7** | **4** | **2** | **4** | **8** | **8** | **3** | **3** | **3** | **4** | **5** | **2** | **2** | **2** | **2** | **2** |
|  | **m_mi_cnv** | **3** | **3** | **4** | **4** | **2** | **6** | **4** | **6** | **7** | **3** | **3** | **3** | **4** | **2** | **6** | **3** | **4** | **3** |
|  | **m_me_cnv** | **3** | **3** | **5** | **2** | **2** | **4** | **2** | **3** | **4** | **3** | **2** | **4** | **5** | **4** | **4** | **6** | **3** | **3** |
|  | **mi_me_cnv** | **3** | **2** | **6** | **4** | **3** | **3** | **2** | **3** | **3** | **3** | **2** | **5** | **5** | **3** | **8** | **7** | **3** | **6** |
|  | **m_mi_me_cnv** | **3** | **3** | **3** | **4** | **5** | **4** | **3** | **6** | **3** | **3** | **3** | **5** | **5** | **2** | **4** | **7** | **6** | **3** |
| **moCluster** | **m_mi** | **4** | **2** | **4** | **4** | **3** | **2** | **6** | **4** | **7** | **2** | **3** | **7** | **2** | **2** | **3** | **2** | **2** | **4** |
|  | **m_me** | **6** | **3** | **2** | **7** | **2** | **8** | **2** | **2** | **3** | **2** | **6** | **3** | **2** | **2** | **8** | **2** | **2** | **2** |
|  | **m_cnv** | **2** | **4** | **7** | **2** | **2** | **6** | **2** | **4** | **8** | **2** | **2** | **4** | **2** | **4** | **5** | **3** | **3** | **8** |
|  | **mi_me** | **2** | **2** | **5** | **2** | **2** | **7** | **2** | **2** | **NA** | **3** | **2** | **8** | **3** | **4** | **6** | **2** | **2** | **2** |
|  | **mi_cnv** | **2** | **2** | **2** | **3** | **2** | **7** | **2** | **2** | **7** | **2** | **4** | **4** | **2** | **2** | **2** | **2** | **2** | **2** |
|  | **me_cnv** | **4** | **3** | **8** | **2** | **3** | **3** | **3** | **4** | **7** | **2** | **5** | **2** | **2** | **2** | **3** | **8** | **2** | **7** |
|  | **m_mi_me** | **2** | **4** | **8** | **3** | **6** | **8** | **2** | **2** | **7** | **7** | **4** | **4** | **3** | **3** | **3** | **2** | **8** | **2** |
|  | **m_mi_cnv** | **3** | **6** | **8** | **2** | **2** | **3** | **8** | **2** | **6** | **2** | **2** | **2** | **2** | **4** | **2** | **2** | **3** | **2** |
|  | **m_me_cnv** | **4** | **7** | **7** | **2** | **2** | **4** | **8** | **8** | **2** | **7** | **2** | **4** | **3** | **4** | **5** | **3** | **2** | **NA** |
|  | **mi_me_cnv** | **3** | **2** | **4** | **2** | **3** | **6** | **7** | **2** | **8** | **2** | **2** | **2** | **2** | **4** | **2** | **2** | **2** | **3** |
|  | **m_mi_me_cnv** | **2** | **2** | **3** | **2** | **6** | **NA** | **4** | **7** | **8** | **3** | **2** | **2** | **2** | **3** | **2** | **2** | **3** | **2** |
| **CIMLR** | **m_mi** | **3** | **4** | **6** | **8** | **7** | **2** | **6** | **8** | **8** | **7** | **3** | **6** | **3** | **8** | **8** | **3** | **8** | **7** |
|  | **m_me** | **3** | **4** | **2** | **2** | **8** | **5** | **7** | **2** | **4** | **3** | **3** | **3** | **5** | **5** | **6** | **5** | **2** | **7** |
|  | **m_cnv** | **6** | **2** | **2** | **5** | **4** | **5** | **8** | **6** | **7** | **5** | **3** | **3** | **5** | **2** | **8** | **7** | **5** | **4** |
|  | **mi_me** | **2** | **2** | **3** | **8** | **2** | **8** | **2** | **6** | **6** | **3** | **4** | **6** | **6** | **2** | **7** | **7** | **4** | **7** |
|  | **mi_cnv** | **2** | **3** | **3** | **2** | **4** | **2** | **3** | **8** | **7** | **4** | **3** | **5** | **6** | **2** | **8** | **7** | **4** | **3** |
|  | **me_cnv** | **8** | **2** | **2** | **3** | **4** | **3** | **2** | **3** | **3** | **8** | **3** | **2** | **2** | **6** | **8** | **8** | **4** | **3** |
|  | **m_mi_me** | **3** | **4** | **6** | **5** | **7** | **5** | **4** | **8** | **8** | **7** | **3** | **6** | **3** | **2** | **8** | **3** | **2** | **3** |
|  | **m_mi_cnv** | **3** | **3** | **3** | **5** | **4** | **2** | **6** | **8** | **7** | **3** | **3** | **8** | **5** | **8** | **8** | **5** | **8** | **8** |
|  | **m_me_cnv** | **3** | **4** | **4** | **6** | **3** | **5** | **7** | **7** | **7** | **8** | **3** | **2** | **5** | **3** | **2** | **8** | **3** | **8** |
|  | **mi_me_cnv** | **3** | **3** | **4** | **8** | **2** | **3** | **3** | **6** | **3** | **8** | **3** | **5** | **8** | **2** | **7** | **7** | **6** | **3** |
|  | **m_mi_me_cnv** | **3** | **3** | **3** | **5** | **8** | **7** | **6** | **2** | **3** | **3** | **3** | **2** | **3** | **2** | **5** | **3** | **2** | **8** |
| **Subtype-GAN** | **m_mi** | **2** | **2** | **2** | **2** | **2** | **2** | **2** | **2** | **2** | **2** | **2** | **2** | **2** | **2** | **2** | **2** | **2** | **2** |
|  | **m_me** | **2** | **2** | **2** | **2** | **2** | **2** | **2** | **2** | **2** | **2** | **2** | **2** | **2** | **2** | **2** | **2** | **2** | **2** |
|  | **m_cnv** | **2** | **2** | **2** | **2** | **2** | **2** | **2** | **2** | **2** | **2** | **2** | **2** | **2** | **2** | **2** | **2** | **2** | **2** |
|  | **mi_me** | **2** | **2** | **2** | **2** | **2** | **2** | **2** | **2** | **2** | **2** | **2** | **2** | **2** | **2** | **2** | **2** | **2** | **2** |
|  | **mi_cnv** | **2** | **2** | **2** | **2** | **2** | **2** | **2** | **2** | **2** | **2** | **2** | **2** | **2** | **2** | **2** | **2** | **2** | **2** |
|  | **me_cnv** | **2** | **2** | **2** | **2** | **2** | **2** | **2** | **2** | **2** | **2** | **2** | **2** | **2** | **2** | **2** | **2** | **2** | **2** |
|  | **m_mi_me** | **2** | **2** | **2** | **2** | **2** | **2** | **2** | **2** | **2** | **2** | **2** | **2** | **2** | **2** | **2** | **2** | **2** | **2** |
|  | **m_mi_cnv** | **2** | **2** | **2** | **2** | **2** | **2** | **2** | **2** | **2** | **2** | **2** | **2** | **2** | **2** | **2** | **2** | **2** | **2** |
|  | **m_me_cnv** | **2** | **2** | **2** | **2** | **2** | **2** | **2** | **2** | **2** | **2** | **2** | **2** | **2** | **2** | **2** | **2** | **2** | **2** |
|  | **mi_me_cnv** | **2** | **2** | **2** | **2** | **2** | **2** | **2** | **2** | **2** | **2** | **2** | **2** | **2** | **2** | **2** | **2** | **2** | **2** |
|  | **m_mi_me_cnv** | **2** | **2** | **2** | **2** | **2** | **2** | **2** | **2** | **2** | **2** | **2** | **2** | **2** | **2** | **2** | **2** | **2** | **2** |

Notations: B-BRCA, C-COAD, KC-KIRC, LA-LUAD, LS-LUSC, A-ACC, KP-KIRP, LI-LIHC, T-THYM. m-mRNA expression, mi-miRNA expression, me-DNA methylation, cnv-copy number variation.

**S2 Table. Suggested *k* of each method using Dataset group #3 Gold Standard Datasets.**

| **Methods** | **Data Combination** | **Complete Datasets** | | **Significant Datasets** | |
| --- | --- | --- | --- | --- | --- |
|  |  | **BRCA** | **COAD** | **BRCA** | **COAD** |
| **iClusterBayes** | **m_mi** | **4** | **3** | **3** | **2** |
|  | **m_me** | **4** | **3** | **3** | **2** |
|  | **m_cnv** | **4** | **3** | **3** | **2** |
|  | **mi_me** | **2** | **3** | **2** | **2** |
|  | **mi_cnv** | **3** | **3** | **3** | **2** |
|  | **me_cnv** | **3** | **2** | **2** | **2** |
|  | **m_mi_me** | **4** | **2** | **3** | **2** |
|  | **m_mi_cnv** | **4** | **3** | **3** | **2** |
|  | **m_me_cnv** | **4** | **2** | **3** | **2** |
|  | **mi_me_cnv** | **4** | **2** | **4** | **2** |
|  | **m_mi_me_cnv** | **4** | **3** | **3** | **2** |
| **SNF** | **m_mi** | **2** | **3** | **2** | **3** |
|  | **m_me** | **2** | **3** | **2** | **3** |
|  | **m_cnv** | **2** | **2** | **2** | **2** |
|  | **mi_me** | **2** | **2** | **2** | **3** |
|  | **mi_cnv** | **2** | **2** | **2** | **2** |
|  | **me_cnv** | **4** | **2** | **3** | **2** |
|  | **m_mi_me** | **2** | **2** | **3** | **2** |
|  | **m_mi_cnv** | **2** | **2** | **3** | **2** |
|  | **m_me_cnv** | **2** | **2** | **2** | **2** |
|  | **mi_me_cnv** | **2** | **3** | **2** | **3** |
|  | **m_mi_me_cnv** | **2** | **2** | **2** | **2** |
| **PINS** | **m_mi** | **22** | **22** | **19** | **25** |
|  | **m_me** | **13** | **9** | **52** | **21** |
|  | **m_cnv** | **14** | **8** | **24** | **2** |
|  | **mi_me** | **15** | **3** | **45** | **2** |
|  | **mi_cnv** | **13** | **5** | **12** | **4** |
|  | **me_cnv** | **7** | **15** | **2** | **2** |
|  | **m_mi_me** | **2** | **8** | **2** | **4** |
|  | **m_mi_cnv** | **2** | **4** | **2** | **2** |
|  | **m_me_cnv** | **5** | **3** | **2** | **3** |
|  | **mi_me_cnv** | **15** | **6** | **2** | **2** |
|  | **m_mi_me_cnv** | **3** | **7** | **2** | **4** |
| **NEMO** | **m_mi** | **3** | **3** | **3** | **3** |
|  | **m_me** | **2** | **3** | **3** | **3** |
|  | **m_cnv** | **4** | **2** | **3** | **2** |
|  | **mi_me** | **7** | **6** | **3** | **3** |
|  | **mi_cnv** | **3** | **2** | **3** | **2** |
|  | **me_cnv** | **6** | **2** | **5** | **2** |
|  | **m_mi_me** | **3** | **3** | **3** | **3** |
|  | **m_mi_cnv** | **3** | **2** | **3** | **2** |
|  | **m_me_cnv** | **2** | **3** | **3** | **3** |
|  | **mi_me_cnv** | **3** | **3** | **3** | **3** |
|  | **m_mi_me_cnv** | **3** | **3** | **3** | **3** |
| **moCluster** | **m_mi** | **4** | **2** | **2** | **2** |
|  | **m_me** | **2** | **6** | **3** | **4** |
|  | **m_cnv** | **3** | **4** | **3** | **7** |
|  | **mi_me** | **2** | **2** | **2** | **2** |
|  | **mi_cnv** | **2** | **8** | **2** | **2** |
|  | **me_cnv** | **8** | **4** | **4** | **5** |
|  | **m_mi_me** | **2** | **6** | **2** | **3** |
|  | **m_mi_cnv** | **3** | **2** | **3** | **2** |
|  | **m_me_cnv** | **3** | **8** | **4** | **2** |
|  | **mi_me_cnv** | **2** | **2** | **4** | **2** |
|  | **m_mi_me_cnv** | **2** | **NA** | **8** | **2** |
| **CIMLR** | **m_mi** | **3** | **3** | **4** | **3** |
|  | **m_me** | **2** | **3** | **7** | **3** |
|  | **m_cnv** | **6** | **3** | **7** | **3** |
|  | **mi_me** | **4** | **2** | **3** | **2** |
|  | **mi_cnv** | **2** | **3** | **2** | **3** |
|  | **me_cnv** | **4** | **6** | **5** | **3** |
|  | **m_mi_me** | **6** | **3** | **3** | **3** |
|  | **m_mi_cnv** | **3** | **3** | **2** | **3** |
|  | **m_me_cnv** | **2** | **3** | **8** | **3** |
|  | **mi_me_cnv** | **2** | **3** | **8** | **3** |
|  | **m_mi_me_cnv** | **3** | **3** | **3** | **3** |
| **Subtype-GAN** | **m_mi** | **2** | **2** | **2** | **2** |
|  | **m_me** | **2** | **2** | **2** | **2** |
|  | **m_cnv** | **2** | **2** | **2** | **2** |
|  | **mi_me** | **2** | **2** | **2** | **2** |
|  | **mi_cnv** | **2** | **2** | **2** | **2** |
|  | **me_cnv** | **2** | **2** | **2** | **2** |
|  | **m_mi_me** | **2** | **2** | **2** | **2** |
|  | **m_mi_cnv** | **2** | **2** | **2** | **2** |
|  | **m_me_cnv** | **2** | **2** | **2** | **2** |
|  | **mi_me_cnv** | **2** | **2** | **2** | **2** |
|  | **m_mi_me_cnv** | **2** | **2** | **2** | **2** |

Notations: m-mRNA expression, mi-miRNA expression, me-DNA methylation, cnv-copy number variation.

**Silhouettes coefficient comparison in the integrated space**

S1 Fig shows the silhouettes coefficient comparison in the integrated space. PINS failed to calculate silhouette in the integrated space because of the unavailability of the integrated matrix. For the results of methods suggested *k*, we found that CIMLR had the highest silhouette coefficient in all cancer datasets and had the best silhouette performance. iClusterBayes, SNF, and moCluster were good and comparable following CIMLR. For the results of all the possible *k*, CIMLR also had the highest silhouette. Other methods were comparable except PFA and Subtype-GAN. Comparing to the silhouette calculated in the original space (Fig 3), we found that the results are inconsistent, especially for CIMLR, LRAcluster, and PFA. The reason can be explained as follows. Because of the characteristic of multi-omic data, high dimensions relative to the small samples, most of the existing methods can not properly separate the samples in the original space. For achieving better clustering results in multi-omics datasets integration, researchers have proposed many effective integration methods, e.g. iClusterBayes, LRAcluster, and PFA, that project features of each omic data into a new integrated space and then cluster samples in this integrated space. From our perspective, such a strategy is good and it represents an important aspect of the integration abilities of methods, but the silhouette may be changed due to the shifted space. Moreover, the dimension of integrated spaces (sample size * feature size which has been greatly reduced and often much smaller than one single omic datatype) are often much lower than the original space (sample size * sum of the feature size of the used omics dataset) which also influences the silhouette coefficient.

**
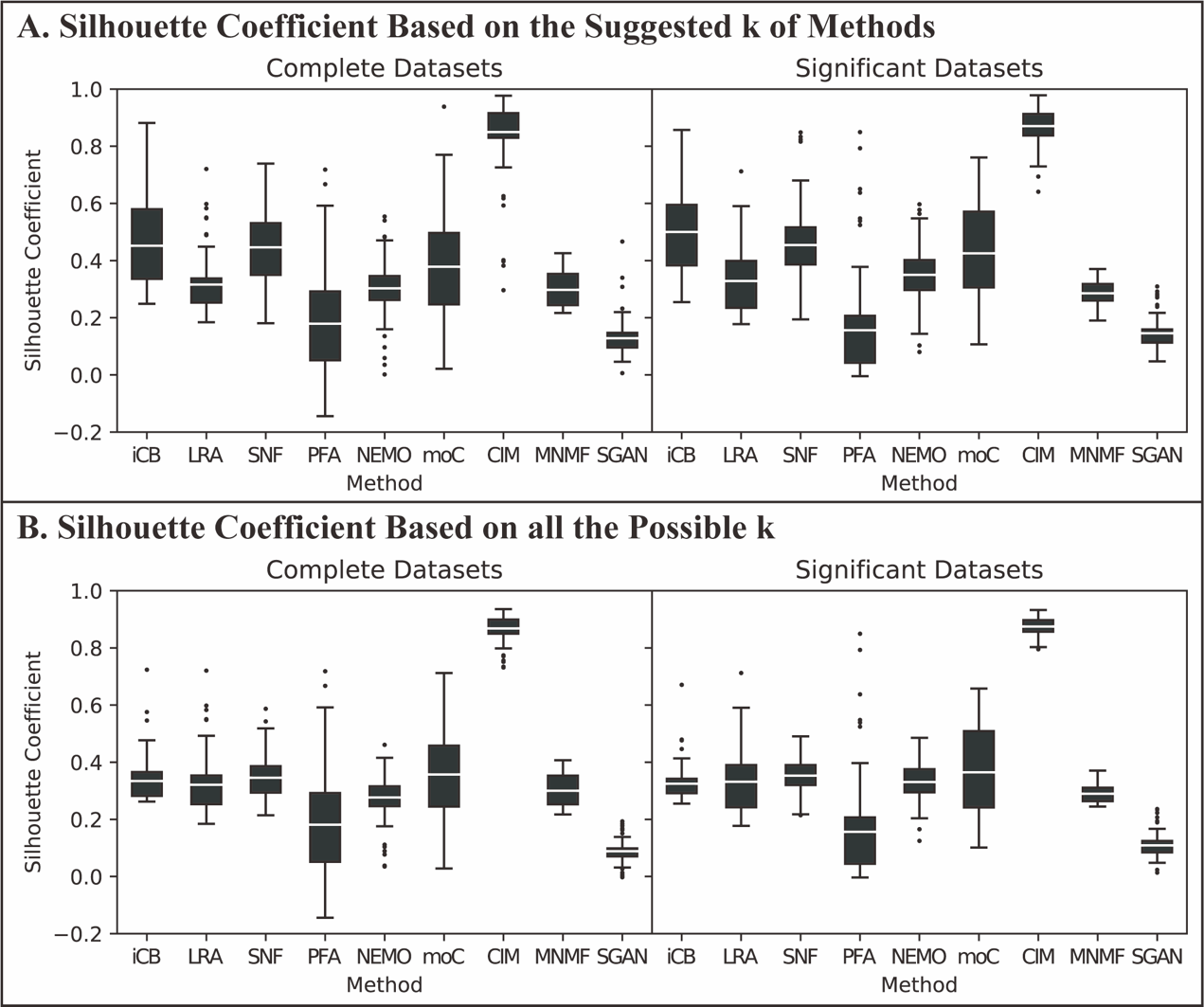
**

**S1 Fig. Silhouette coefficient comparison based on integrated space of Dataset group #1 Nine-cancer Datasets.** We use “iCB”, “LRA”, “moC”, “CIM”, “MNMF” and “SGAN” to represent iClusterBayes, LRAcluster, moCluster, CIMLR, MultiNMF, and Subtype-GAN, respectively. (A) Silhouette coefficient based on the suggested *k* of methods. We set *k*-max as 8 and let each method suggest the best *k*. Each of the 11 data points in a box represents a silhouette coefficient of the subtyping results based on the method suggested *k* obtained by the corresponding method using one of the 11 possible combinations of data types. (B) Silhouette coefficient based on all the possible *k*. Each of the 11 data points in a box represents the average silhouette coefficient of the subtyping results from *k*=2 to 8 obtained by the corresponding method using one of the 11 possible combinations of data types.
